# Relationships between plant traits, soil properties and carbon fluxes differ between monocultures and mixed communities in temperate grassland

**DOI:** 10.1111/1365-2745.13160

**Published:** 2019-03-25

**Authors:** Jonathan R. De Long, Benjamin G. Jackson, Anna Wilkinson, William J. Pritchard, Simon Oakley, Kelly E. Mason, Jörg G. Stephan, Nicholas J. Ostle, David Johnson, Elizabeth M. Baggs, Richard D. Bardgett

**Affiliations:** ^1^ School of Earth and Environmental Sciences The University of Manchester Manchester UK; ^2^ Department of Terrestrial Ecology Netherlands Institute of Ecology Wageningen The Netherlands; ^3^ The Global Academy of Agriculture and Food Security, The Royal (Dick) School of Veterinary Studies University of Edinburgh Midlothian UK; ^4^ Centre for Ecology & Hydrology, Lancaster Environment Centre Bailrigg UK; ^5^ Department of Ecology Swedish University of Agricultural Sciences Uppsala Sweden; ^6^ Lancaster Environment Centre Lancaster University Lancaster UK

**Keywords:** above‐ground–below‐ground linkages, biodiversity, carbon, ecosystem function, net ecosystem exchange, nitrogen, plant functional traits, soil microbial communities

## Abstract

1. The use of plant traits to predict ecosystem functions has been gaining growing attention. Above‐ground plant traits, such as leaf nitrogen (N) content and specific leaf area (SLA), have been shown to strongly relate to ecosystem productivity, respiration and nutrient cycling. Furthermore, increasing plant functional trait diversity has been suggested as a possible mechanism to increase ecosystem carbon (C) storage. However, it is uncertain whether below‐ground plant traits can be predicted by above‐ground traits, and if both above‐ and below‐ground traits can be used to predict soil properties and ecosystem‐level functions.
2. Here, we used two adjacent field experiments in temperate grassland to investigate if above‐ and below‐ground plant traits are related, and whether relationships between plant traits, soil properties and ecosystem C fluxes (i.e. ecosystem respiration and net ecosystem exchange) measured in potted monocultures could be detected in mixed field communities.
3. We found that certain shoot traits (e.g. shoot N and C, and leaf dry matter content) were related to root traits (e.g. root N, root C:N and root dry matter content) in monocultures, but such relationships were either weak or not detected in mixed communities. Some relationships between plant traits (i.e. shoot N, root N and/or shoot C:N) and soil properties (i.e. inorganic N availability and microbial community structure) were similar in monocultures and mixed communities, but they were more strongly linked to shoot traits in monocultures and root traits in mixed communities. Structural equation modelling showed that above‐ and below‐ground traits and soil properties improved predictions of ecosystem C fluxes in monocultures, but not in mixed communities on the basis of community‐weighted mean traits.
4. *Synthesis*. Our results from a single grassland habitat detected relationships in monocultures between above‐ and below‐ground plant traits, and between plant traits, soil properties and ecosystem C fluxes. However, these relationships were generally weaker or different in mixed communities. Our results demonstrate that while plant traits can be used to predict certain soil properties and ecosystem functions in monocultures, they are less effective for predicting how changes in plant species composition influence ecosystem functions in mixed communities.

## 1 INTRODUCTION

The role plant traits play in driving ecosystem processes has been the focus of much recent research (Díaz et al., 2016; Faucon, Houben, & Lambers, 2017; Kimball et al., 2016). For example, key plant traits and the proportion of a plant community consisting of species with ‘slow’ versus ‘fast’ functional traits (Díaz et al., 2016; Reich, 2014; Wright et al., 2004) influence soil functions, such as decomposition (Fortunel et al., 2009; Quested, Eriksson, Fortunel, & Garnier, 2007) and nutrient cycling (Fortunel et al., 2009; Grigulis et al., 2013). Furthermore, increasing plant functional trait diversity has been proposed as a potential mechanism by which to increase carbon (C) allocation and storage below‐ground (De Deyn, Cornelissen, & Bardgett, 2008). Root traits have been linked with below‐ground C inputs (Guyonnet, Cantarel, Simon, & Haichar, 2018), decomposition rates (Freschet, Aerts, & Cornelissen, 2012; Smith, Woodin, Pakeman, Johnson, & Wal, 2014), soil C storage (Lange et al., 2015) and soil physical properties (Gould, Quinton, Weigelt, De Deyn, & Bardgett, 2016), which have wider implications for soil functioning. Despite these advances, there remains considerable uncertainty as to which plant functional traits (above‐ground and/or below‐ground) best predict soil properties and ecosystem processes. It also remains unknown if these plant trait‐soil linkages extend from individual plants to mixed plant communities in real world contexts. Furthermore, whether plant traits–soil linkages can improve our ability to understand the factors controlling processes such as net ecosystem exchange and ecosystem respiration, which determine C gain or loss from an ecosystem, remains understudied.

There has been growing interest in determining whether or not an analogue to the leaf economic spectrum exists for roots and if below‐ground traits can be used alongside above‐ground traits to better predict soil properties and ecosystem functions (Bardgett, Mommer, & de Vries, 2014; Kramer‐Walter et al., 2016; Roumet et al., 2016). For instance, Roumet et al. (2016) used 74 plant species from three biomes to show that root nitrogen (N) concentration and specific root length (SRL) were positively correlated with root respiration, while root dry matter content (RDMC) and the lignin‐to‐N ratio were negatively correlated to mass remaining after decomposition. Furthermore, Pérez‐Ramos et al. (2012) showed in an experiment in Mediterranean rangeland that leaf and root traits responded similarly to N limitation (i.e. they became more conservative), indicating coordination between above‐ and below‐ground resource acquisition strategies, which lends support to the existence of a root economic spectrum. In a study of 66 New Zealand tree species, however, Kramer‐Walter et al. (2016) found that although above‐ground traits were strongly related to growth rate, below‐ground traits were not. Furthermore, in a study comparing above‐ and below‐ground traits across 34 tree species, no evidence for a root economic spectrum was detected; instead, root traits were more strongly determined by phylogenetic relatedness (Valverde‐Barrantes, Smemo, & Blackwood, 2015). These reported relationships between above‐ and below‐ground traits appear to vary between ecosystems highlighting the remaining uncertainties in our understanding of shoot–root trait linkages.

Although past studies have led to advances in our understanding of linkages between plant traits and ecosystem functions, they have mostly investigated them using artificially constructed plant communities or environmental gradients. Artificially constructed communities based on random selections of species typically include very low species and functional diversity (Fischer et al., 2016; Milcu et al., 2014; Roscher et al., 2004; Spehn et al., 2005; Tilman et al., 2001). Yet, under natural conditions, we know that species generally assemble non‐randomly and when very low levels of diversity do occur (e.g. through environmental extremes), they consist of highly non‐random sets of species with distinct functional attributes (Wardle, 2016). Furthermore, environmental gradients typically involve concomitant changes to both environmental factors and the plant community. This makes it difficult to tease apart plant community responses to the gradient from their effects on soil properties and ecosystem processes (Grigulis et al., 2013; Kichenin, Wardle, Peltzer, Morse, & Freschet, 2013; Legay et al., 2014; Manning et al., 2015; Sundqvist, Giesler, & Wardle, 2011). As such, our understanding of the importance of plant traits in driving soil properties and ecosystem functioning is limited to specific contexts. Therefore, experimental designs that seek to understand linkages between plant traits and soil properties in realistic communities are needed (Wardle, 2016).

**Figure 1:**
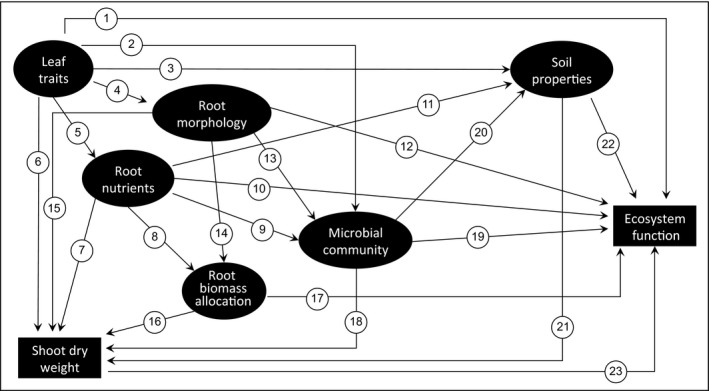
A priori path model for ecosystem carbon fluxes in the monoculture and community experiments. Arrows indicate causal directed relationships between latent variables (LVs) (ellipses). LVs reflected by only one measured variable are shown as boxes. Leaf economic traits, in particular leaf N content, scale with plant photosynthetic and respiratory capacity (**1**) (Reich, 2014), while root economic and architectural traits may contribute to the partitioning of C below‐ground affecting soil respiration (**10, 12**) (De Deyn et al., 2008). Leaf and root traits often show coordinated variation in stoichiometry and tissue density (**5**) (Freschet et al., 2010), but traits relating to root morphology can be a secondary, and potentially independent dimension of root trait variation (**4**) (Kramer‐Walter et al., 2016) as we observed in this study (see Figure S2). The fast–slow spectrum can drive relative growth rates (**6, 7, 8**) (Reich, 2014; Wright et al., 2004) and root morphology may drive patterns of C allocation above‐ and below‐ground (**14**,** 15**) (Guyonnet et al., 2018; Lange et al., 2015) and thus collectively standing biomass above‐ and below‐ground, while relative investment of growth above‐ versus below‐ground can drive root to shoot ratios (**16**) (Kimball et al., 2016). The fast–slow leaf spectrum can drive the relative importance of fungal versus bacterial dominated energy channels below‐ground (**2, 9**) (Legay et al., 2014), while patterns of plant C allocation below‐ground in terms of root length and root diameter may impact on root colonization rates and patterns of C exudation with consequences for microbial community structure (**13**) (Lange et al., 2015). The fast–slow spectrum of trait variation (**3**,** 11**) (Reich, 2014; Wright et al., 2004) and the stoichiometry and structure of the microbial community (**20**) (de Vries & Bardgett, 2016; de Vries et al., 2012) can influence soil carbon and nitrogen stocks, inorganic and organic nitrogen availability and mineralization rates, which in turn can impact carbon allocation below‐ground (**18**) (De Deyn et al., 2008), plant growth rates and biomass accumulation (**21**) (Reich, 2014; Wright et al., 2004). Plant above‐ground biomass can drive both photosynthetic and respiratory rates above‐ground (**23**) (Grigulis et al., 2013; Reich, 2014; Wright et al., 2004), while below‐ground plant biomass allocation and the stoichiometry and structure of the microbial community can influence below‐ground respiration rates (**17, 19**) (Grigulis et al., 2013; Roumet et al., 2016). Soil C and N stocks, inorganic and organic N availability and mineralization rates can influence rates of biological activity in soil and thus below‐ground respiration rates (**22**) (Grigulis et al., 2013)

To address these uncertainties, we tested in potted monocultures of a broad range of temperate grassland plant species whether leaf and root traits are related, and identified which traits best predict soil properties and ecosystem carbon fluxes, including ecosystem respiration and net ecosystem exchange. Furthermore, we tested whether relationships between traits and soil properties found in monocultures could also be detected in mixed plant communities in the field under more natural conditions. First, we hypothesized that leaf and root traits related to C and N allocation would show consistent relationships in both monocultures and mixed communities, because traits related to C and N show consistent relationships across entire plants (i.e. leaves and roots) (Bardgett et al., 2014; Freschet, Cornelissen, Logtestijn, & Aerts, 2010; Reich, 2014). Second, we expected that, in both monocultures and mixed communities, leaf and root traits would predict soil properties related to C cycling because plant traits can influence soil microbial communities (Legayet al., 2014, 2016; Orwin et al., 2010) and soil abiotic properties (Reich, 2014; Wright et al., 2004), which strongly influence ecosystem C cycling. Third, we hypothesized that leaf and root traits, and soil properties in both monoculture and mixed field communities (i.e. using community‐weighted mean (CWM) trait values) would predict ecosystem processes (i.e. C fluxes). This is because plant traits related to C and N allocation have been shown to strongly and consistently influence C cycling across spatial scales and contrasting ecosystems (Funk et al., 2017). Using both leaf and root traits in tandem with soil properties could provide a powerful opportunity to improve our predictive capacity and management of C cycling in grasslands (Faucon et al., 2017; Kimball et al., 2016). To this end, we constructed an a priori model (Figure 1) depicting proposed relationships between leaf and root traits, soil properties and ecosystem C fluxes. We then used this model to test if these relationships were similar in both potted monocultures and mixed field communities.

We tested these hypotheses using a pot‐based monoculture study grown under field conditions, which included 25 common grassland species from three plant functional groups (PFGs) (i.e. grasses, forbs and legumes) and covering a wide range of above‐ and below‐ground trait variation. In parallel, we set up a grassland community experiment manipulating plant species and functional diversity. Factorial combinations of plant species from the same functional groups used in the monoculture experiment were added to extant grassland communities to create a gradient of plant species and functional diversity. The functional group addition treatments were applied within the same field specifically so that all the plots started with similar plant communities, soil properties and environmental conditions, which enabled us to test for the influence of the functional attributes of the plant community on soil properties and ecosystem processes. This was done with minimal disturbance to the extant plant communities, so as to more closely mimic management practices for the conservation and restoration of botanical diversity in UK grasslands (Bullock et al., 2011; Pywellet al., 2002, 2007). We focused on soil properties related to C cycling, including nutrient availability and microbial community structure, as well as ecosystem respiration and net ecosystem exchange, which determine the ability of grasslands to act as net C sources or sinks (Trumper, Programme, & UNEP, GRID‐Arendal & Centre, U. W. C. M., 2009).

## 2 MATERIALS AND METHODS

### 2.1 Study site and experimental design

Both experiments were conducted within Ingleborough National Nature Reserve in northern England (54° 11' 38.7" N, 2° 20' 54.4” W) as described by Leff et al. (2018). Plant communities at both sites are *Lolium perenne*–*Cynosurus cristatus*‐dominated grasslands (MG6; Rodwell, 1998) and are situated at 300 m a.s.l. Annual average daily minimum and maximum temperatures between 1981 and 2010 were 4.3°C and 10.5°C, respectively; average annual precipitation was 1550 mm (www.metoffice.gov.uk).

The potted monoculture experiment was conducted outside in a fenced enclosure at Colt Park Meadows. Monocultures were constructed in May 2013 from polypropylene pots (38 × 38 × 40 cm) filled with 10 cm of rinsed gravel and 20 cm sieved topsoil; a design consistent with other studies (Bardgett et al., 2006; Harrison & Bardgett, 2010; Legay et al., 2016; Orwin, Ostle, Wilby, & Bardgett, 2014). Topsoil was collected from the adjacent meadow and is a clayey brown earth over limestone bedrock (pH ~ 5.8; 8.9 C%; 0.92 N%) from the Malham series of Eutric Endoleptic Cambisols (Cranfield University, 2018). The meadow is lightly grazed by sheep (≤1.7 individuals/h) and cattle (≤0.3 individuals/h) during the winter and spring, with hay harvested each summer and a light dressing of well‐rotted farmyard manure applied in early spring in some years. Twenty‐five native grassland plant species were selected for the monoculture experiment (Table S1). The species selected represent the three dominant plant functional groups (PFG) typical of British mesotrophic meadow grasslands (Rodwell, 1998; Smith et al., 2008). The PFGs used here (i.e. grasses, forbs and legumes) have been widely used in experiments because plants from the same functional group have similar effects and responses to ecosystem processes and environmental conditions respectively (Hooper et al., 2005). Furthermore, these species encompassed a breadth of leaf trait variation (e.g. SLA: 12–45 mm^2^/mg; shoot N: 7.8–44.7 mg N g^−1^; Table S1), comparable to one of the longest running experiments exploring the role of plant diversity in ecosystem functioning (SLA: 8–38 mg N g^−1^; shoot N: 15–50 mm^2^/mg; Roscher et al., 2018). Plants were germinated in a greenhouse from commercial seed (Emorsgate Seeds, King's Lynn, Norfolk, UK). Then, 30 seedlings of each species were transplanted into pots to produce monocultures of 25 species (*n* = 4 replicates per species or 100 pots total); such seedling densities are comparable with previous studies (Legay et al., 2016; Orwin et al., 2014). The planted monocultures were arranged in a randomized block design within four blocks, with one of each species per block and weeded regularly during the growing season. The planted monocultures grew outside under ambient conditions for three growing seasons (2013–2015). To emulate normal winter grazing of the meadow and the summer hay crop, all above‐ground biomass in the monocultures was harvested from the pots in May 2014 and 2015, and September 2014, respectively. Plant and soil material were collected from all monocultures in July 2015 at peak plant biomass. Five fully emerged leaves from at least three individuals per pot were clipped and refrigerated. In addition, from each pot, a 6.8‐cm diameter soil core was taken, sieved to 4 mm and stored at 4ºC. All roots not passing through the sieve were retained and stored at 4°C before being washed free of soil prior to analysis for root traits and estimation of below‐ground biomass (see below). Although some roots may have been lost through the sieving process, all root measurements were taken from samples that passed through a 4‐mm sieve, meaning that comparisons between experiments are robust. Finally, all above‐ground biomass was harvested from the pots, oven dried and weighed.

The community experiment was conducted 2 km from the monoculture experiment at Selside Shaw (54° 10’ 47.9" N, 2° 20' 11.1” W). Experimental plots were established in temperate grassland of similar management and vegetation to Colt Park. The soils are part of the same Malham Series of Eutric Endoleptic Cambisols as at Colt Park; a clayey brown earth (pH ~ 5.7; 4.9 C%; 0.46 N%) and prior to the establishment of the experiment the site received either a light dressing of farmyard manure or 125 kg/ha of 20:10:10 NPK fertilizer each year. In 2012, 36‐m^2^ experimental plots were assigned to one of seven PFG addition treatments (grasses, forbs, legumes and their factorial combinations) or as control plots to which no PFGs were added. This yielded eight treatment combinations replicated five times in a fully randomized block design, giving a total of 40 plots. Based on the species complement of typical species‐rich meadow communities of northern pastures (UK National Vegetation Classification MG3b; Rodwell, 1998), intact plant communities within each plot were planted with greenhouse‐grown seedlings in 2013–2015 and seeded in 2014–2015 (see Tables S2, S3 and S4 in Supporting Information for details). The target functional groups were both seeded and planted into the intact grassland in order to rapidly maximize establishment and minimize disturbance, thereby generating a hybrid between restoration practices and more manipulative experiments (i.e. those that till under the existent community and reseed a target community). Furthermore, there was no pretreatment applied to the field (e.g. tillage or herbicide) in order to replicate restoration practices largely based on seeding previously used for theses grasslands (Smith et al., 2008). Plant species composition was measured in May 2016 in ten 100‐cm^2^ quadrats systematically distributed in each plot and averaged to provide an estimate of percentage cover of each species in each plot. Plant species composition and above‐ground biomass was also measured in each of the base rings used to measure CO_2_ exchange (see below) in order to directly link the plant species composition and relative abundances, as well as CWM traits to gas fluxes. Plant species composition within the gas rings was representative of the plant species composition of the entire plot.

### 2.2 Plant functional traits

Above‐ and below‐ground plant functional traits were measured in both the monoculture and community experiments. This was done because it is well known that environmental conditions (i.e. plants grown in pots in monocultures vs. those grown in mixed communities in the field) can have major effects on traits (Siefert et al., 2015), which could further impact on ecosystem functions (Albert, Grassein, Schurr, Vieilledent, & Violle, 2011; de Bello et al., 2011). In July 2015, intact, undamaged, fully expanded leaves were collected from at least three individual plants from each monoculture and refrigerated. We used an EPSON flatbed scanner and WinRhizo software (Regent Instruments Inc., Canada) to calculate specific leaf area (SLA) and leaf dry matter content (LDMC) exactly as outlined in Cornelissen et al. (2003). Finally, each leaf was crushed and analysed for C and N (Elementar Vario EL element analyser, Hanau, Germany). Leaf fibre lignin (LFL) was estimated on another leaf sample using an ANKOM 200 fibre analyser (ANKOM, Macedon, NY). Values from the three leaves taken per pot were then averaged to generate a single replicate value for each leaf trait measurement for each pot.

In July 2015, root samples were collected from a 6.8‐cm diameter soil core from each of the pots. An EPSON flatbed scanner and WinRhizo (Regent Instruments, Quebec, Canada) were used to calculate SRL and RDMC exactly as outlined in Cornelissen et al. (2003). Each bulk root sample was ground (Retsch Ball Mill MM 400, Haan, Germany) and analysed for C and N and root fibre lignin (RFL), as specified above.

In August 2016, five undamaged, fully expanded leaves were collected from each of 15 species in a subset of 10 plots (the control unamended plots and those amended with species from all three PFGs) so that leaf traits could be compared between the monocultures and the mixed plant communities. The 15 species collected accounted on average for over 80% of the vegetation cover in each plot (Table S5) and all of these species (with the exception of *Poa trivialis*) were also represented in the monoculture experiment (Table S1). In October 2016, root samples were collected from five 4.5‐cm diameter cores of 10 cm depth taken from each of the 40 plots across all treatments immediately adjacent to the gas rings (see below). Because root systems of individual plant species are highly intertwined, it was not possible to gain a true CWM measure for individual root traits. Instead, we took a composite measure of root traits from the mixed root systems collected from each soil core (Legay et al., 2014). Leaf and root samples were analysed for trait values in the same way as the monoculture experiment. Leaf trait data from the community plots was then combined with the vegetation survey data (see above) from within the gas flux base rings (Figure S1) to calculate CWM trait values for each plot following Garnier et al. (2007).

### 2.3 Soil nutrients and microbial properties

Soils were collected from the monocultures (one 6.8‐cm diameter core) and the community experiment (three randomly sampled 2‐cm diameter cores per plot) in July 2015 and June 2016, respectively. In the community experiment, soil samples were collected adjacent to the gas flux base rings (see below). This was done so that we could continue to take measurements from within the gas rings without disturbing the soil and plant communities. Five grams (fresh weight) of soil was extracted with 25 ml of 1 M KCl and shaken for 1 hr. Extracts were frozen at −18ºC until analysis for available NH_4_‐N, NO_3_‐N, total inorganic nitrogen (TIN), dissolved organic nitrogen and total nitrogen (N) on a Seal AA3 Segmented Flow Multi‐chemistry analyser (Mequon, WI, USA). Microbial C and N were measured using chloroform‐fumigation (Brookes, Landman, Pruden, & Jenkinson, 1985). The bases Mg, K, Al were leached from a subsample of soil with ammonium acetate and analysed by optical emission spectroscopy with inductively coupled plasma excitation (Blakemore & Dyer, 1959). Microbial community structure, based on phospholipid fatty acids (PLFA), was analysed on an additional subsample of soil for each monoculture and community plot (Bligh & Dyer, 1959; White, Davis, Nickels, King, & Bobbie, 1979). PLFAs were extracted from freeze‐dried soil (Frostegård, Tunlid, & Bååth, 1991), as modified by Buyer and Sasser (2012) and analysed on a gas chromatograph (Agilent 7890A Gas chromatograph, Santa Clara, CA). Abundance of PLFAs is expressed in µg/g dry weight soil. PLFAs were assigned as indicators of fungal and bacterial abundance (De Deyn et al., 2011) and C18:2ω6,9 only was used for saprophytic fungi (Frostegård, Tunlid, & Bååth, 2011). We also calculated the ratios of fungal to bacterial markers and Gram‐positive to Gram‐negative bacterial markers.

### 2.4 CO_2_ measurements

Measurements of CO_2_ fluxes were made over 120‐s intervals with a PP systems EGM4 portable IRGA (infrared gas analyser) coupled to custom‐built chambers in a closed loop gas circuit. For the monoculture experiment, chambers were constructed with liteglaze acrylic sheeting (92% light transmission) fixed to a polypropylene frame. The chambers sealed against the outer rim of the pots, enclosing both plants and soil and had a headspace volume of 0.038 m^3^ (Orwin et al., 2014). For the community experiment, in each sampling plot a 30‐cm diameter, 10‐cm high permanent base ring was fitted in place to a depth of 5 cm in spring 2014; care was taken to minimize disturbance and to avoid severance of large plant roots. The chambers were made of translucent domed plastic cloches, 30 cm diameter and 35 cm height, fitted to a polypropylene ring sealing against the base ring with a headspace volume of 0.039 m^3^ (Ward, Bardgett, McNamara, Adamson, & Ostle, 2007). We used the dark and light flux method to estimate ecosystem respiration and net ecosystem exchange fluxes, respectively (Ward et al., 2007). For the monocultures, flux measurements were made in the final growing season on four dates in 2015: 12 May and 6, 16 and 30 June. For the community experiment, flux measurements were made during spring and summer of 2016: 18 May, 7 June, 5, 26 and 27 July, and 2, 4, 11 and 23 August. In all cases, flux measurements were taken between 10:00 and 16:00 hr and paired with measures of air temperature, soil moisture and ambient light conditions (i.e. photosynthetically active radiation) that were taken simultaneously at the time of each flux measurement.

### 2.5 Statistical analyses

First, exploratory principal component analyses (PCA) were performed to identify patterns of shoot and root trait covariation as well as covariation in soil biotic and abiotic properties in the monoculture and community experiments. Two components from each of the trait PCAs and three components from each of the soil PCAs were retained based on parallel analyses using the *nFactors* package in R. Relationships between variables loading strongly on the retained components were then examined by pairwise linear regressions to assess links between shoot and root traits, and traits and soil properties in both the monoculture and community experiments. Additionally, standard major axis regressions (SMA) were performed to assess the strength of the relationships between the principle components from the PCAs of shoot and root traits and the respective components of variation in soil properties from the two experiments. A SMA is a form of model 2 regression that accounts for the uncertainty in both variables by minimizing the errors in both x and y directions.

Second, the effects of the PFG addition treatments on plant species diversity and relative abundance, and the soil microbial communities (i.e. PLFAs) in the community experiment, were assessed by multivariate analysis of variance (permANOVA) using the *adonis2* function in the *vegan* package in R. Bray–Curtis dissimilarities were calculated and corrected for negative eigenvalues before constraining permutations (999) to account for the blocking design. In addition, the effects of the functional group addition treatments on the functional attributes of the plant communities were tested by one‐way ANOVA for individual shoot and root traits and collectively across all traits by calculating functional diversity indices (Villéger, Mason, & Mouillot, 2008).

**Figure 2:**
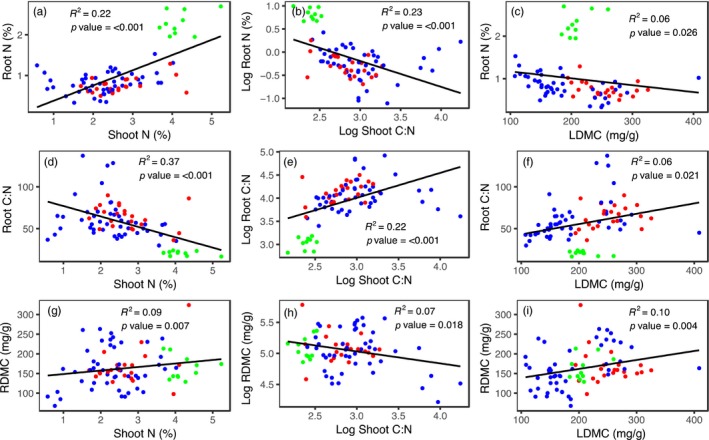
Relationships between selected shoot and root traits of 25 temperate grassland species grown in monoculture under field conditions (a‐i). Plant functional groups are shown as red = grass, blue = forb and green = legume. Full regression matrix of relationships between shoot and root traits for the monoculture experiment is shown in Table S7 [Colour figure can be viewed at wileyonlinelibrary.com]

**Figure 3:**
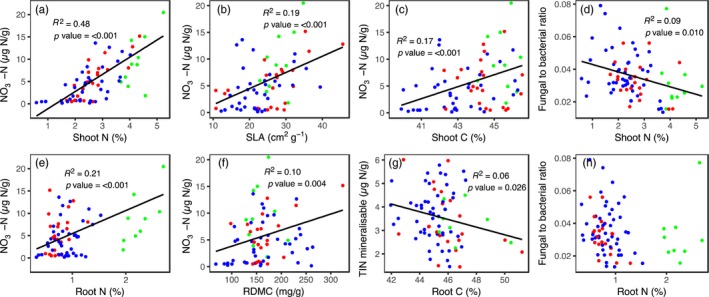
Relationships between shoot and root traits with selected soil properties of 25 temperate grassland species grown in monoculture under field conditions (a‐h). Plant functional groups are shown as red = grass, blue = forb and green = legume. SLA—specific leaf area, RDMC—root dry matter content, TIN—total inorganic nitrogen (NO_3_‐N and NH_4_‐N). Full regression matrix of relationships between plant traits and soil properties for the monoculture experiment is shown in Table S8 [Colour figure can be viewed at wileyonlinelibrary.com]

**Figure 4:**
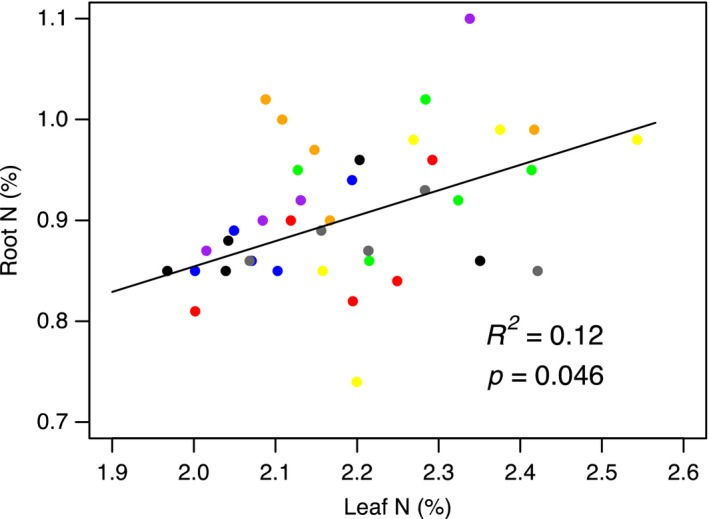
Relationship between community‐weighted mean shoot N and root N from the 40 mixed community plots. Treatments are shown as black = control, red = grasses, blue = forbs, green = legumes, purple = grasses + forbs, orange = grasses + legumes, yellow = forbs + legumes, grey = grasses + forbs + legumes. Full regression matrix of shoot traits and root trait relationships is shown in Table S9 [Colour figure can be viewed at wileyonlinelibrary.com]

**Figure 5:**
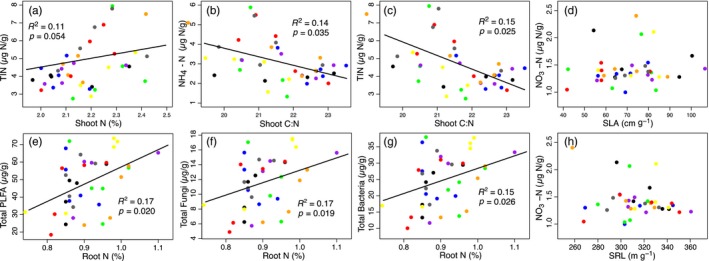
Relationships between leaf and root traits with selected soil properties in the 40 mixed community plots (a‐h). Treatments are shown as black = control, red = grasses, blue = forbs, green = legumes, purple = grasses + forbs, orange = grasses + legumes, yellow = forbs + legumes, grey = grasses + forbs + legumes. SRL TIN—total inorganic nitrogen (NO_3_‐N and NH_4_‐N). Full regression matrix of trait—soil relationships is shown in Table S10 [Colour figure can be viewed at wileyonlinelibrary.com]

We constructed partial least squares path models (PLS‐PMs) to test direct and indirect controls of leaf and root traits, soil properties and soil microbial attributes on above‐ground biomass production and mean values of instantaneous ecosystem respiration and net ecosystem exchange (averaged over the growing season) in both the monoculture and the community experiments with net ecosystem exchange normalized relative to PAR for each date. A common a priori model structure (Figure 1) was established for the monoculture and community experiments based on our hypotheses and theoretical knowledge of trait–soil–process linkages. We chose PLS‐PM (Chin & Dibbern, 2010), a form of structural equation modelling, because we had many measured variables indicative of broader biotic and abiotic patterns that could be included in latent variables (LVs) and relatively low numbers of observations given the complexity of our a priori model structure (Hair, Hult, Ringle, Sarstedt, & Thiele, 2017). Measured variables were chosen as indicative of LVs based on the loadings on PCAs axes (Figures S2, S3, S4, S5) and pairwise relationships (Figures 2, 3, 4, 5 and Tables S6, S7, S8, S9, S10). For the community experiment, all shoot trait values used in the model were input as CWMs. Rescaled data were fitted to the a priori model using the *plspm* package in R using Stone–Geisser's predictive relevance as the primary evaluation criterion for model fit. See supporting information for all relevant details related to model construction, simplification and validation processes (Methods S1).

## 3 RESULTS

### 3.1 Linkages between plant traits, soil properties and C fluxes in monocultures

Exploratory PCA and SMA analyses of the trait data from the monoculture experiment revealed primary axes of leaf and root trait variation related most strongly to tissue N concentrations; these axes scaled positively with each other (*R* ^2^ = 0.19, *p* ≤ 0.001; Figure S5a; Table S6). The secondary leaf and root axes were related to LDMC and root morphology (i.e. SRL, root diameter), respectively (Figure S2a,b), whereas the second leaf axis scaled weakly with both the first (*R* ^2^ = 0.11, *p* = 0.005) and second (*R* ^2^ = 0.06, *p* = 0.034) root trait axes (Table S6). Pairwise regressions between traits showed that shoot N, shoot C:N and LDMC were all significantly related to root N, root C:N and RDMC (Figure 2), but varied in their strength of association from weak (e.g. LDMC vs. RDMC; *R* ^2^ = 0.10, *p* = 0.004) to moderate (e.g. shoot N vs. root C:N; *R* ^2^ = 0.37, *p* ≤ 0.001). Several additional pairwise relationships between leaf and root traits were observed (Table S7).

Leaf and root traits showed significant relationships with soil abiotic and biotic properties (Table S8). However, more and stronger relationships were detected between shoot traits and soil properties than roots traits. Both shoot and root N were related to soil NO_3_‐N (Figure 3a,b), with shoot N explaining more than double the variation (*R* ^2^ = 0.48, *p* ≤ 0.001) in soil NO_3_‐N compared to roots (*R* ^2^ = 0.21, *p* ≤ 0.001). Additionally, all of the leaf traits were related in some way to the soil microbial community. Notably, shoot N (Figure 3d), shoot C:N and SLA were significantly, although weakly, related to the fungal to bacterial ratio of the soils. In contrast, no root traits were related to the soil microbial communities (Table S8). This was reflected in the scaling of the primary axes of both shoot and root trait variation being related to the secondary axis of variation in the soil properties, along which TIN loaded most strongly (Table S6).

**Figure 6:**
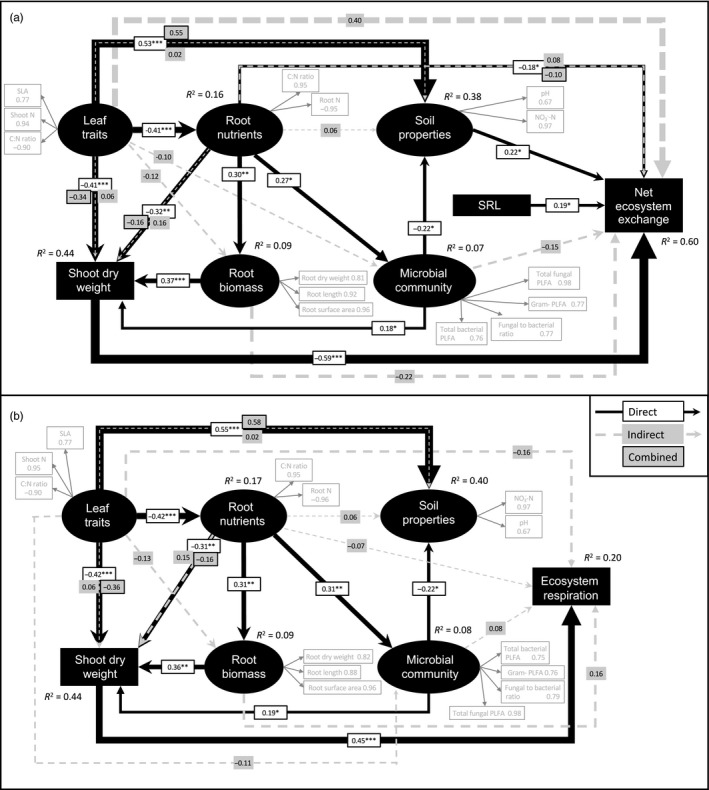
Partial least‐squares path models showing the relationships between leaf and root traits, soil properties, microbial community attributes, above‐ground biomass and (a) net ecosystem exchange (GoF index 0.470) and (b) ecosystem respiration (GoF index 0.418) in monocultures of 25 temperate grassland species. Reflective LVs (black ovals) are indicated by measured variables (grey boxes) with their respective loadings shown. The width of the arrows indicates the strength of the causal relationships supplemented by standardized path coefficients (*p < 0.05; **p < 0.01; ***p < 0.001). *R* ^2^ values indicate the explained variance of response variables. To meet the requirements of unidimensionality, the indicator variables in (a) shoot C:N, root N and net ecosystem exchange, and in (b) shoot C:N and root N were multiplied by negative one. See Methods S1 for details on model selection procedure

The final path models for the monoculture experiment showed that leaf and root traits explained significant variation in net ecosystem exchange (Figure 6a, *R* ^2^ = 0.60) and moderate variation in ecosystem respiration (Figure 6b, *R* ^2^ = 0.20). Furthermore, SLA, shoot N and shoot C to N ratios collectively made moderate, indirect (but no direct) contributions to net ecosystem exchange (path coefficient: 0.40). Specifically, plants with lower SLA and shoot N resulted in greater shoot dry weight and in turn higher net uptake of CO_2_ (i.e. net ecosystem exchange, see Methods S1). Shoot dry weight had a direct negative effect on net ecosystem exchange (−0.59). In addition, plants with lower SRL weakly increased net ecosystem exchange (0.19). The microbial community and root nutrients were not significant predictors of net ecosystem exchange. Furthermore, SLA, shoot N and the shoot C to N ratios collectively made an overall weak contribution to ecosystem respiration (−0.16) through three indirect paths. First, plants with higher SLA and shoot N resulted in lower shoot dry weight (−0.42), which directly led to lower ecosystem respiration (0.45). Second, higher SLA and shoot N were associated with higher root N (−0.42). Soil abiotic properties were not related to ecosystem respiration. Plants with higher N content (i.e. root nutrients) were associated with lower root biomass (0.31; direction of relationship inverted for technical reasons), but neither the root economic traits nor root biomass were associated with ecosystem respiration. Finally, plants with higher SLA and shoot N were associated with soils with higher NO_3_‐N concentrations (Figure 6b, *R* ^2^ = 0.40), but soil nutrients did not predict ecosystem respiration.

### 3.2 Linkages between plant traits, soil properties and C fluxes in mixed field communities

PermANOVA revealed that the functional group addition treatments in the community experiment significantly changed the composition of the plant communities (*R* ^2^ = 0.23, *F* = 1.39, *p* ≤ 0.001). The treatments successfully increased the percentage cover and species richness of forbs and legumes, but not of grasses (Figure S6). Relative to the control plots, forb cover increased by approximately 10%–18% in the “Grass + Forb” treatment (Figure S6c) and forb richness nearly doubled in all the treatments where forbs were added (Figure S6d). Both legume cover and richness doubled in all the treatments where legumes were added (Figure S6e,f), while grass cover and richness were not significantly affected by any of the treatments (Figure S6a,b). Across all species, mean values of functional traits for the individual species grown in monoculture were similar to the mean trait values of the same species collected from the community (notable exceptions include SLA and SRL, which were shifted higher and lower in the mixed communities compared to the monocultures, respectively; Tables S11, S12). Plant functional traits were more variable (i.e. high coefficients of variation) between the species grown in monoculture than compared to the CWMs for the same traits in the community.

The functional group addition treatments altered CWM shoot N in the community plots (*F* = 2.76, *p* = 0.024) with the “Forb + Legume” treatment having ~ 10% higher shoot N than the “Forb” treatment (Figure S7). Furthermore, CWM SLA at the plot level varied between 25.79 and 41.73 cm^2^/g, CWM shoot C to N ratio varied from 18.96 to 23.52, and SRL varied between 41.78 and 106.47 m/g (Table S12). Although functional diversity indices were not affected by the functional group addition treatments, they varied across plots: functional richness (FRic) and functional diversity (FDiv) across the 40 plots ranged from 0.03 to 1.84 (CV 64.82%) and 0.66 to 0.93 (CV 7.98%), respectively (Table S12).

Of the leaf and root traits measured in the community experiment, only leaf N and root N were positively related (*R* ^2^ = 0.12, *p* = 0.046; Figure 4, and Table S9). This pairwise relationship underpinned the significant covariation (*R* ^2^ = 0.10, *p* = 0.047; Table S6) between the primary axes of shoot and root trait variation from the community experiment (Figure S5b). Plant community traits also showed weak relationships with soil properties in the community experiment (Figure 5; Table S10), with more relationships observed between root traits and soil properties than for shoot traits. The CWM shoot C to N ratio was negatively to TIN and NH_4_‐N (Figure 5b,c). In contrast, root N was not related to soil concentrations of N (Table S10). PermANOVA revealed that the PFG addition treatments did not result in significant differences in the composition of the soil microbial communities (*R* ^2^ = 0.08, *p* = 0.572). However, root N was significantly, but weakly, related to most soil microbial community properties including total PLFAs, total fungal PLFAs, total bacterial PLFAs, Gram‐positive and Gram‐negative bacterial PLFAs, and microbial biomass N (Figure 5e,f,g). SRL and root diameter were unrelated to any soil biotic or abiotic properties.

**Figure 7:**
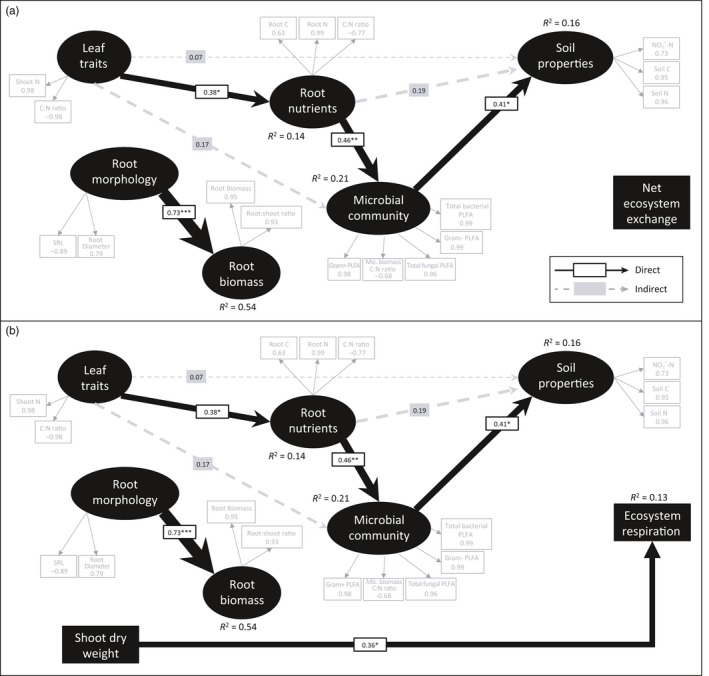
Partial least‐squares path models showing the relationships between leaf and root traits, soil properties, microbial community attributes, above‐ground biomass and (a) net ecosystem exchange (GoF index 0.4623) and (b) ecosystem respiration (GoF index 0.4383) in mixed grassland communities. Reflective LVs (black ovals) are indicated by measured variables (grey boxes) with their respective loadings shown. The width of the arrows indicates the strength of the causal relationships supplemented by standardized path coefficients (*p < 0.05; **p < 0.01; ***p < 0.001). *R* ^2^ values indicate the explained variance of response variables. To meet the requirements of unidimensionality, the indicator variables in (a) shoot C:N, root C:N and microbial C:N and in (b) shoot C:N, root C, microbial C:N and total bacterial PLFA were multiplied by negative one. See Methods S1 for details on model selection procedure

The PLS‐PMs for the community experiment did not explain net ecosystem exchange (Figure 7a). However, plant communities with higher shoot dry weight had higher mean rates of ecosystem respiration (0.36; *R* ^2^ = 0.13). Additionally, in both community models, shoot N concentration and the shoot C to N ratio collectively affected soil properties (0.07, Figure 7) indirectly via their collective influence of root C, root N and the root C to N ratios (0.19), and in turn via their influence on the soil microbial community (0.17). Plant communities with higher leaf N had higher root N concentrations, which was associated with soil microbial communities with lower microbial biomass C to N ratios and greater fungal and bacterial abundance. These microbial community attributes resulted in soils with higher total C and N and higher NO_3_‐N concentrations. Finally, PLS‐PMs that attempted to predict above‐ground productivity (i.e. shoot dry weight) were not significant, nor were models that attempted to predict net ecosystem exchange and ecosystem respiration using PFG in place of resource economic traits (data not shown).

## 4 DISCUSSION

Here, we assessed relationships between leaf and root traits, soil properties and ecosystem C fluxes in temperate grassland, and found similar patterns of trait covariation above‐ and below‐ground in monocultures and mixed communities. In both monocultures and communities, we found that leaf traits were generally weak predictors of root traits, but that some leaf and root traits were significant predictors of soil properties. We found that plant traits were moderate predictors of ecosystem C fluxes (i.e. ecosystem respiration and net ecosystem exchange) in monocultures, primarily indirectly through their influence on shoot biomass. However, in mixed species plots, net ecosystem exchange was unrelated to plant traits and soil properties, and ecosystem respiration was best predicted by shoot biomass. These findings show that while relationships between plant traits, soil properties, and C fluxes in monocultures and mixed communities shared some commonalities (e.g. between tissue N and labile soil N), they were relatively few and often weak. Below, we discuss our findings and their significance for making predictions about plant trait relationships to ecosystem processes.

In partial support of our first hypothesis, we found the primary axes of shoot and root trait covariation were underpinned by tissue C and N concentrations in both the monocultures and communities. In contrast to prior studies (e.g. Freschet et al., 2010, Craine, Lee, Bond, Williams, & Johnson, 2005), only 15% and 10% of the variation in the root axis was explained by the leaf axis in the monocultures and communities, respectively. Such weak linkages between shoot and root traits could be due to the narrower functional range of species considered (i.e. all species were herbaceous). Interestingly, leaf N was orthogonal to SLA in the field communities, but these two traits were positively related in the monocultures (Figure S2a,c). This may be because in the monocultures, individual species showed a more coordinated response between above‐ and below‐ground organs due to a lack of interspecific competition or other confounding factors (e.g. more complex soil abiotic and biotic conditions) present in the mixed field communities. Nonetheless, the coordination of leaf and root C and N in both monocultures and communities supports prior observations of integrated leaf and root resource strategies and a potential whole‐plant economic spectrum (Freschet et al., 2010; Pérez‐Ramos et al., 2012; Reich, 2014). However, in both the monocultures and the communities, SRL and root diameter were not related to leaf and root N, forming a second independent dimension of root trait variation. A similar secondary dimension of root trait variation was recently observed in a broad range of tree species (Kramer‐Walter et al., 2016) and Ma et al. (2018) found that at the global level root traits do not follow the same patterns observed in above‐ground traits. Together with our observations, these results support the hypothesis that root traits are multidimensional (Weemstra et al., 2016), reflecting the greater potential range of root trait combinations necessary to enhance plant fitness under different environmental conditions (Bardgett, 2017; Laliberté, 2017; Laughlin, 2014; Zemunik, Turner, Lambers, & Laliberté, 2015).

Our second hypothesis was partially supported: some relationships between plant traits and soil properties in monocultures and mixed communities were similar. In monocultures, we found that shoot and root C and N, as well as SLA and RDMC, were moderate predictors of soil inorganic N, while shoot traits such as SLA and shoot N predicted the soil fungal to bacterial ratio, which has been linked to soil properties, such as N retention, in grassland soils (Bardgett & McAlister, 1999; Orwin et al., 2010; de Vries et al., 2012). In communities, shoot N predicted soil concentrations of inorganic N, as in the monocultures, but there was a stronger link between root traits and soil properties. For example, bacterial and fungal PLFAs were better predicted by root N in the communities than in the monocultures (Figure 5f, g). This finding may be due to competition for soil resources in the field (as compared to the monocultures), potentially leading to tighter associations between root traits and the soil microbial community (Hortal et al., 2017). Furthermore, the PLS‐PMs showed contrasts in the relationships between plant traits and soil abiotic and biotic properties between monocultures and communities. In the monocultures, links were detected between leaf traits and soil abiotic properties, as well as between root nutrients and the microbial community (Figure 6). This supports other work that has shown strong links between plant traits and soil properties in monocultures (Orwin et al., 2010; Thion et al., 2016) and in mesocosm experiments (Milcu et al., 2014; de Vries & Bardgett, 2016). In field communities, leaf traits were poor predictors of soil properties, but the link between root nutrients and the soil microbial community was maintained (Figure 7). This is likely because plant root traits associated with C and N cycling influence nutrient availability for soil microbial communities in grasslands (Legay et al., 2014).

Overall, root traits were weak predictors of soil abiotic properties in communities. This could be for a number of reasons. For instance, the relatively low variation in soil properties within the field site may have led to species with similar traits occupying the available niche space (Kumordzi et al., 2015). Indeed, previous studies that have identified relationships between root traits and soil properties at a community level have done so across sites with strong divergence in plant traits and soil properties (Freschet et al., 2010; Legay et al., 2014; Pérez‐Ramos et al., 2012). It is also possible that root traits may have become more similar in the field due to biotic interactions generating interspecific trait convergence (Gubsch et al., 2011), or that the influence of root traits may have been limited to the rhizosphere, whereas we sampled the bulk soil; this may have masked certain linkages between root traits and soil properties. Less commonly reported traits, including phenological traits, might capture differences in temporal resource use (Ebeling et al., 2014) that may allow plants to compete despite overlap between other functional traits. Our findings show that despite linkages between shoot and root traits and soil properties in monocultures, many of these relationships do not translate to parallel influences on soil properties at the community level, which highlights the need to better understand the mechanisms underpinning these relationships.

Our third hypothesis was also partially supported: plant traits and soil properties helped predict C fluxes in monocultures, but not in mixed communities. When grown in monoculture, leaf and root traits either directly or indirectly predicted net ecosystem exchange and ecosystem respiration, and shoot biomass was also a strong predictor. The relationships detected between plant traits and C fluxes were likely due to the strong conditioning effects plants exerted on the soil in the pots over the 2 years of the monoculture experiment. In contrast, in the communities, net ecosystem exchange and above‐ground productivity were not predicted by plant traits or functional groups, and the best predictor of ecosystem respiration was shoot biomass. In both monocultures and communities, soil microbial community did not explain C fluxes, even though plant identity can control soil microbial community composition in this grassland (Leff et al., 2018). The lack of influence of the soil microbial community on C fluxes is possibly due to high functional redundancy in microbial communities (Persiani, Maggi, Montalvo, Casado, & Pineda, 2008). Interestingly, shoot biomass was a strong predictor of ecosystem respiration in both the monocultures and mixed communities, and of net ecosystem exchange in monocultures. This may be because the total biomass of the plant community is the overriding driving force behind grassland C fluxes, given that plants are the primary source of C fixation via photosynthesis.

The absence of relationships between plant traits, soil properties and ecosystem C fluxes in our field communities contrasts with other studies that have shown plant traits to predict grassland soil properties and ecosystem function (Grigulis et al., 2013; Legay et al., 2016; Manning et al., 2015; Milcu et al., 2014). However, these studies either considered plant communities with artificially high variation in trait values (Milcu et al., 2014) or communities that were highly variable due to strongly contrasting environmental gradients (Grigulis et al., 2013; Legay et al., 2016; Manning et al., 2015). The lack of relationships between CWM traits, soil properties, and ecosystem C fluxes in our study may reflect limited changes to the functional attributes of the plant communities generated by the PFG addition treatments, where only CWM shoot N was affected. Nevertheless, variation of some traits across individual plots was relatively high (e.g. SLA 25.79 to 41.73 mm^2^/mg; SRL 41.7–106.4 m/g) and comparable to the extent of trait variation reported in prior studies of grasslands across larger spatial scales. For example, de Vries et al. (2012) observed CWM SLA to vary from 17.6 to 35.1 mm^2^/mg for a range of grassland types across England, while Grigulis et al. (2013) recorded a range in CWM SLA from 5 to 25 mm^2^/mg across montane grasslands in three geographically distinct locations in Europe. Furthermore, across a 2000‐km regional transect in Inner Mongolia, Cheng, Chu, Chen, Bai, and Niu (2016) measured a breadth of variation in CWM SRL from 1 to 91 m/g. As such, the gradients of trait variation in our field communities were reasonably representative of those found in other studies at much larger scales, thereby enabling us to test our hypotheses on links between above‐ and below‐ground traits, soil properties and ecosystem C fluxes.

Despite high variation in some plant traits at the plot level, the limited extent of the functional variation across field plant community treatments probably reflects the relatively short timeframe of our experiment. It likely also reflects that our experimental plots were not pretreated (i.e. tilled or sprayed with herbicide) prior to seeding and planting, which is consistent with biodiversity restoration practice in agriculturally improved grasslands (Smith et al., 2008). Furthermore, variation in factors such plant age, plant–plant competition and soil properties across plots may have masked relationships between traits and ecosystem function (Funk et al., 2017), as might also legacy effects of previous land management on soil properties (i.e. fertilization regimes). Our experimental plots had species richness ranging from 16 to 42, with a mean of 27 species per plot (Figure S6), which is well above the median number of species typically found in manipulative grassland experiments (Roscher et al., 2004; Spehn et al., 2005; Tilman et al., 2001). Therefore, the number of species present in this system may be beyond a critical threshold where any further changes in plant trait diversity can impact on ecosystem processes. This finding has important implications for restoration practices where there is a growing consensus that relationships between functional traits and ecosystem processes can guide restoration of grassland (Zirbel, Bassett, Grman, & Brudvig, 2017; Zuo et al., 2016). However, much of this evidence concerns restoration from fields that had markedly different management or land‐use histories (e.g. crop production or dunes). By contrast, our study shows that manipulation of plant species diversity and/or functional traits is unlikely to facilitate restoration of extant grassland to promote ecosystem services related to C cycling, at least in the short term, and highlights the need to develop more effective restoration tools for these circumstances.

In conclusion, despite detecting some similar relationships between leaf and root traits and soil properties in monocultures and mixed species plots, relationships were fewer, weaker and often different, in mixed communities. In the field communities, we also found that many of the most widely measured plant functional traits were not related to observed variation in key components of ecosystem CO_2_ flux, specifically net ecosystem exchange and ecosystem respiration. This finding suggests that broad scale patterns linking functional traits to ecosystem processes might not hold at local scales (Messier, Lechowicz, McGill, Violle, & Enquist, 2017). This could be due to a high overlap in plant trait functioning that confers functional redundancy within our grassland site, and/or it is possible that more time was needed before changes in plant community trait composition result in significant changes to ecosystem C fluxes. However, our results also demonstrate that realistic species diversity manipulations at local scales do not necessarily lead to rapid changes in soil properties that are typically seen in more artificial plant diversity grassland experiments both in the field and in mesocosms (Milcu et al., 2014; Roscher et al., 2004; Tilman et al., 2001). Collectively, our results from a single grassland demonstrate that while plant traits can be used to predict certain soil properties and ecosystem functions in monocultures, they may be less effective for predicting how changes in plant species composition influence soil properties and ecosystem C fluxes in mixed communities.

## AUTHORS’ CONTRIBUTIONS

R.D.B. initiated and designed the study in collaboration with E.M.B., N.J.O. and D.J. A.W. led the establishment of the experiments, and J.R.D.L., B.G.J., A.W., W.J.P., S.O. and K.E.M. collected the data; J.R.D.L., B.G.J. and J.G.S. analysed the data; J.R.D.L. and B.G.J. led the writing of the manuscript, with contributions from all co‐authors.

## Data Availability

Data available from the Dryad Digital Repository: https://doi.org/10.5061/dryad.gh41n3j (Jackson, 2019).

## References

[jec13160-bib-0001] Albert, C. H. , Grassein, F. , Schurr, F. M. , Vieilledent, G. , & Violle, C. (2011). When and how should intraspecific variability be considered in trait‐based plant ecology? Perspectives in Plant Ecology Evolution and Systematics, 13, 217–225.

[jec13160-bib-0002] Bardgett, R. D. (2017). Plant trait‐based approaches for interrogating belowground function. *Biology and Environment* . Proceedings of the Royal Irish Academy, 117B, 1–13.

[jec13160-bib-0003] Bardgett, R. D. , & McAlister, E. (1999). The measurement of soil fungal: Bacterial biomass ratios as an indicator of ecosystem self‐regulation in temperate meadow grasslands. Biology and Fertility of Soils, 29, 282–290. 10.1007/s003740050554

[jec13160-bib-0004] Bardgett, R. D. , Mommer, L. , & De Vries, F. T. (2014). Going underground: Root traits as drivers of ecosystem processes. Trends in Ecology & Evolution, 29, 692–699. 10.1016/j.tree.2014.10.006 25459399

[jec13160-bib-0005] Bardgett, R. D. , Smith, R. S. , Shiel, R. S. , Peacock, S. , Simkin, J. M. , Quirk, H. , & Hobbs, P. J. (2006). Parasitic plants indirectly regulate below‐ground properties in grassland ecosystems. Nature, 439, 969–972. 10.1038/nature04197 16495998

[jec13160-bib-0007] Bligh, E. G. , & Dyer, W. J. (1959). A rapid method of total lipid extraction and purification. Canadian Journal of Biochemistry and Physiology, 37, 911–917. 10.1139/y59-099 13671378

[jec13160-bib-0008] Brookes, P. C. , Landman, A. , Pruden, G. , & Jenkinson, D. S. (1985). Chloroform fumigation and the release of soil‐nitrogen ‐ a rapid direct extraction method to measure microbial biomass nitrogen in soil. Soil Biology & Biochemistry, 17, 837–842. 10.1016/0038-0717(85)90144-0

[jec13160-bib-0009] Bullock, J. M. , Jefferson, R. G. , Blackstock, T. H. , Pakeman, R. J. , Emmet, B. A. , Pywell, R. F. , … Silvertown, J. (2011). Chapter 6: semi‐natural grasslands in the UK national ecosystem assessment technical report. Cambridge: UNEP‐WCMC.

[jec13160-bib-0010] Buyer, J. S. , & Sasser, M. (2012). High throughput phospholipid fatty acid analysis of soils. Applied Soil Ecology, 61, 127–130. 10.1016/j.apsoil.2012.06.005

[jec13160-bib-0011] Cheng, J. , Chu, P. , Chen, D. , Bai, Y. , & Niu, S. (2016). Functional correlations between specific leaf area and specific root length along a regional environmental gradient in Inner Mongolia grasslands. Functional Ecology, 30, 985–997. 10.1111/1365-2435.12569

[jec13160-bib-0012] Chin, W. W. , & Dibbern, J. (2010). Handbook of partial least squares concepts, methods and applications. Heilderberg: Springer.

[jec13160-bib-0013] Cornelissen, J. h. c. , Lavorel, S. , Garnier, E. , Díaz, S. , Buchmann, N. , Gurvich, D. e. , … Poorter, H. (2003). A handbook of protocols for standardised and easy measurement of plant functional traits worldwide. Australian Journal of Botany, 51, 335–380. 10.1071/BT02124

[jec13160-bib-0014] Craine, J. M. , Lee, W. G. , Bond, W. J. , Williams, R. J. , & Johnson, L. C. (2005). Environmental constraints on a global relationship among leaf and root traits of grasses. Ecology, 86, 12–19. 10.1890/04-1075

[jec13160-bib-0015] Cranfield University . (2018). The soils guide. UK: Cranfield University Retrieved from www.landis.org.uk

[jec13160-bib-0016] de Bello, F. , Lavorel, S. , Albert, C. H. , Thuiller, W. , Grigulis, K. , Dolezal, J. , … Lepš, J. (2011). Quantifying the relevance of intraspecific trait variability for functional diversity. Methods in Ecology and Evolution, 2, 163–174. 10.1111/j.2041-210X.2010.00071.x

[jec13160-bib-0017] De Deyn, G. B. , Cornelissen, J. H. C. , & Bardgett, R. D. (2008). Plant functional traits and soil carbon sequestration in contrasting biomes. Ecology Letters, 11, 516–531. 10.1111/j.1461-0248.2008.01164.x 18279352

[jec13160-bib-0018] De Deyn, G. B. , Shiel, R. S. , Ostle, N. J. , McNamara, N. P. , Oakley, S. , Young, I. , … Bardgett, R. D. (2011). Additional carbon sequestration benefits of grassland diversity restoration. Journal of Applied Ecology, 48, 600–608. 10.1111/j.1365-2664.2010.01925.x

[jec13160-bib-0019] de Vries, F. T. , & Bardgett, R. D. (2016). Plant community controls on short‐term ecosystem nitrogen retention. New Phytologist, 210, 861–874. 10.1111/nph.13832 26749302 PMC4981912

[jec13160-bib-0020] de Vries, F. T. , Bloem, J. , Quirk, H. , Stevens, C. J. , Bol, R. , & Bardgett, R. D. (2012). Extensive management promotes plant and microbial nitrogen retention in temperate grassland. PLoS ONE, 7, 1–12. 10.1371/journal.pone.0051201 PMC3515579 23227252

[jec13160-bib-0021] Díaz, S. , Kattge, J. , Cornelissen, J. H. C. , Wright, I. J. , Lavorel, S. , Dray, S. , … Gorné, L. D. (2016). The global spectrum of plant form and function. Nature, 529, 167–173. 10.1038/nature16489 26700811

[jec13160-bib-0022] Ebeling, A. , Pompe, S. , Baade, J. , Eisenhauer, N. , Hillebrand, H. , Proulx, R. , … Weisser, W. W. (2014). A trait‐based experimental approach to understand the mechanisms underlying biodiversity–ecosystem functioning relationships. Basic and Applied Ecology, 15, 229–240. 10.1016/j.baae.2014.02.003

[jec13160-bib-0023] Faucon, M.‐P. , Houben, D. , & Lambers, H. (2017). Plant functional traits: Soil and ecosystem services. Trends in Plant Science, 22, 385–394. 10.1016/j.tplants.2017.01.005 28209328

[jec13160-bib-0024] Fischer, F. M. , Wright, A. J. , Eisenhauer, N. , Ebeling, A. , Roscher, C. , Wagg, C. , … Pillar, V. D. (2016). Plant species richness and functional traits affect community stability after a flood event. Philosophical Transactions of the Royal Society B‐Biological Sciences, 371, 1–8. 10.1098/rstb.2015.0276 PMC4843697 27114578

[jec13160-bib-0025] Fortunel, C. , Garnier, E. , Joffre, R. , Kazakou, E. , Quested, H. , Grigulis, K. , … Zarovali, M. (2009). Leaf traits capture the effects of land use changes and climate on litter decomposability of grasslands across Europe. Ecology, 90, 598–611. 10.1890/08-0418.1 19341132

[jec13160-bib-0026] Freschet, G. T. , Aerts, R. , & Cornelissen, J. H. C. (2012). A plant economics spectrum of litter decomposability. Functional Ecology, 26, 56–65. 10.1111/j.1365-2435.2011.01913.x

[jec13160-bib-0027] Freschet, G. T. , Cornelissen, J. H. C. , van Logtestijn, R. S. P. , & Aerts, R. (2010). Evidence of the 'plant economics spectrum' in a subarctic flora. Journal of Ecology, 98, 362–373. 10.1111/j.1365-2745.2009.01615.x

[jec13160-bib-0028] Frostegård, Å. , Tunlid, A. , & Bååth, E. (1991). Microbial biomass measured as total lipid phosphate in soils of different organic content. Journal of Microbiological Methods, 14, 151–163. 10.1016/0167-7012(91)90018-L

[jec13160-bib-0029] Frostegård, A. , Tunlid, A. , & Bååth, E. (2011). Use and misuse of PLFA measurements in soils. Soil Biology & Biochemistry, 43, 1621–1625. 10.1016/j.soilbio.2010.11.021

[jec13160-bib-0030] Funk, J. L. , Larson, J. E. , Ames, G. M. , Butterfield, B. J. , Cavender‐Bares, J. , Firn, J. , … Wright, J. (2017). Revisiting the Holy Grail: Using plant functional traits to understand ecological processes. Biological Reviews, 92, 1156–1173. 10.1111/brv.12275 27103505

[jec13160-bib-0031] Garnier, E. , Lavorel, S. , Ansquer, P. , Castro, H. , Cruz, P. , Dolezal, J. , … Zarovali, M. p. (2007). Assessing the effects of land‐use change on plant traits, communities and ecosystem functioning in grasslands: A standardized methodology and lessons from an application to 11 European sites. Annals of Botany, 99, 967–985. 10.1093/aob/mcl215 17085470 PMC2802906

[jec13160-bib-0032] Gould, I. J. , Quinton, J. N. , Weigelt, A. , De Deyn, G. B. , & Bardgett, R. D. (2016). Plant diversity and root traits benefit physical properties key to soil function in grasslands. Ecology Letters, 19, 1140–1149. 10.1111/ele.12652 27459206 PMC4988498

[jec13160-bib-0033] Grigulis, K. , Lavorel, S. , Krainer, U. , Legay, N. , Baxendale, C. , Dumont, M. , … Clément, J.‐C. (2013). Relative contributions of plant traits and soil microbial properties to mountain grassland ecosystem services. Journal of Ecology, 101, 47–57. 10.1111/1365-2745.12014

[jec13160-bib-0034] Gubsch, M. , Buchmann, N. , Schmid, B. , Schulze, E. D. , Lipowsky, A. , & Roscher, C. (2011). Differential effects of plant diversity on functional trait variation of grass species. Annals of Botany, 107, 157–169. 10.1093/aob/mcq220 21068024 PMC3002477

[jec13160-bib-0035] Guyonnet, J. P. , Cantarel, A. A. M. , Simon, L. , & Haichar, F. E. Z. (2018). Root exudation rate as functional trait involved in plant nutrient‐use strategy classification. Ecology and Evolution, 8, 8573–8581. 10.1002/ece3.4383 30250724 PMC6144958

[jec13160-bib-0036] Hair, J. F. , Hult, G. T. M. , Ringle, C. M. , Sarstedt, M. , & Thiele, K. O. (2017). Mirror, mirror on the wall: A comparative evaluation of composite‐based structural equation modeling methods. Journal of the Academy of Marketing Science, 45, 616–632. 10.1007/s11747-017-0517-x

[jec13160-bib-0037] Harrison, K. A. , & Bardgett, R. D. (2010). Influence of plant species and soil conditions on plant‐soil feedback in mixed grassland communities. Journal of Ecology, 98, 384–395. 10.1111/j.1365-2745.2009.01614.x

[jec13160-bib-0038] Hooper, D. U. , Chapin, F. S. , Ewel, J. J. , Hector, A. , Inchausti, P. , Lavorel, S. , … Wardle, D. A. (2005). Effects of biodiversity on ecosystem functioning: A consensus of current knowledge. Ecological Monographs, 75, 3–35. 10.1890/04-0922

[jec13160-bib-0039] Hortal, S. , Lozano, Y. m. , Bastida, F. , Armas, C. , Moreno, J. l. , Garcia, C. , & Pugnaire, F. i. (2017). Plant‐plant competition outcomes are modulated by plant effects on the soil bacterial community. Scientific Reports, 7, 1–9. 10.1038/s41598-017-18103-5 29259319 PMC5736699

[jec13160-bib-0040] Jackson, B. (2019). Data from: Relationships between plant traits, soil properties and carbon fluxes differ between monocultures and mixed communities in temperate grassland. Dryad Digital Repository, 10.5061/dryad.gh41n3j PMC6617750 31341333

[jec13160-bib-0041] Kichenin, E. , Wardle, D. A. , Peltzer, D. A. , Morse, C. W. , & Freschet, G. T. (2013). Contrasting effects of plant inter‐ and intraspecific variation on community‐level trait measures along an environmental gradient. Functional Ecology, 27, 1254–1261. 10.1111/1365-2435.12116

[jec13160-bib-0042] Kimball, S. , Funk, J. L. , Spasojevic, M. J. , Suding, K. N. , Parker, S. , & Goulden, M. L. (2016). Can functional traits predict plant community response to global change? Ecosphere, 7, 1–18.

[jec13160-bib-0043] Kramer‐Walter, K. R. , Bellingham, P. J. , Millar, T. R. , Smissen, R. D. , Richardson, S. J. , & Laughlin, D. C. (2016). Root traits are multidimensional: Specific root length is independent from root tissue density and the plant economic spectrum. Journal of Ecology, 104, 1299–1310. 10.1111/1365-2745.12562

[jec13160-bib-0044] Kumordzi, B. B. , de Bello, F. , Freschet, G. T. , Le Bagousse‐Pinguet, Y. , Leps, J. , & Wardle, D. A. (2015). Linkage of plant trait space to successional age and species richness in boreal forest understorey vegetation. Journal of Ecology, 103, 1610–1620. 10.1111/1365-2745.12458

[jec13160-bib-0045] Laliberté, E. (2017). Below‐ground frontiers in trait‐based plant ecology. New Phytologist, 213, 1597–1603. 10.1111/nph.14247 27735077

[jec13160-bib-0046] Lange, M. , Eisenhauer, N. , Sierra, C. A. , Bessler, H. , Engels, C. , Griffiths, R. I. , … Gleixner, G. (2015). Plant diversity increases soil microbial activity and soil carbon storage. Nature Communications, 6, 1–8. 10.1038/ncomms7707 25848862

[jec13160-bib-0047] Laughlin, D. C. (2014). The intrinsic dimensionality of plant traits and its relevance to community assembly. Journal of Ecology, 102, 186–193. 10.1111/1365-2745.12187

[jec13160-bib-0048] Leff, J. W. , Bardgett, R. D. , Wilkinson, A. , Jackson, B. G. , Pritchard, W. J. , De Long, J. R. , … Fierer, N. (2018). Predicting the structure of soil communities from plant community taxonomy, phylogeny, and traits. The ISME Journal, 12, 1794–1805. 10.1038/s41396-018-0089-x 29523892 PMC6004312

[jec13160-bib-0049] Legay, N. , Baxendale, C. , Grigulis, K. , Krainer, U. , Kastl, E. , Schloter, M. , … Lavorel, S. (2014). Contribution of above‐ and below‐ground plant traits to the structure and function of grassland soil microbial communities. Annals of Botany, 114, 1011–1021. 10.1093/aob/mcu169 25122656 PMC4171078

[jec13160-bib-0050] Legay, N. , Lavorel, S. , Baxendale, C. , Krainer, U. , Bahn, M. , Binet, M.‐N. , … Bardgett, R. D. (2016). Influence of plant traits, soil microbial properties, and abiotic parameters on nitrogen turnover of grassland ecosystems. Ecosphere, 7, 1–17. 10.1002/ecs2.1448

[jec13160-bib-0051] Ma, Z. , Guo, D. , Xu, X. , Lu, M. , Bardgett, R. D. , Eissenstat, D. M. , … Hedin, L. O. (2018). Evolutionary history resolves global organization of root functional traits. Nature, 555, 94–97. 10.1038/nature25783 29466331

[jec13160-bib-0052] Manning, P. , de Vries, F. T. , Tallowin, J. R. B. , Smith, R. , Mortimer, S. R. , Pilgrim, E. S. , … Bardgett, R. D. (2015). Simple measures of climate, soil properties and plant traits predict national‐scale grassland soil carbon stocks. Journal of Applied Ecology, 52, 1188–1196. 10.1111/1365-2664.12478

[jec13160-bib-0053] Messier, J. , Lechowicz, M. J. , McGill, B. J. , Violle, C. , & Enquist, B. J. (2017). Interspecific integration of trait dimensions at local scales: The plant phenotype as an integrated network. Journal of Ecology, 105, 1775–1790. 10.1111/1365-2745.12755

[jec13160-bib-0054] Milcu, A. , Roscher, C. , Gessler, A. , Bachmann, D. , Gockele, A. , Guderle, M. , … Roy, J. (2014). Functional diversity of leaf nitrogen concentrations drives grassland carbon fluxes. Ecology Letters, 17, 435–444. 10.1111/ele.12243 24393400

[jec13160-bib-0055] Orwin, K. H. , Buckland, S. M. , Johnson, D. , Turner, B. L. , Smart, S. , Oakley, S. , & Bardgett, R. D. (2010). Linkages of plant traits to soil properties and the functioning of temperate grassland. Journal of Ecology, 98, 1074–1083. 10.1111/j.1365-2745.2010.01679.x

[jec13160-bib-0056] Orwin, K. H. , Ostle, N. , Wilby, A. , & Bardgett, R. D. (2014). Effects of species evenness and dominant species identity on multiple ecosystem functions in model grassland communities. Oecologia, 174, 979–992. 10.1007/s00442-013-2814-5 24213721

[jec13160-bib-0057] Pérez‐Ramos, I. M. , Roumet, C. , Cruz, P. , Blanchard, A. , Autran, P. , & Garnier, E. (2012). Evidence for a 'plant community economics spectrum' driven by nutrient and water limitations in a Mediterranean rangeland of southern France. Journal of Ecology, 100, 1315–1327. 10.1111/1365-2745.12000

[jec13160-bib-0058] Persiani, A. M. , Maggi, O. , Montalvo, J. , Casado, M. A. , & Pineda, F. D. (2008). Mediterranean grassland soil fungi: Patterns of biodiversity, functional redundancy and soil carbon storage. Plant Biosystems, 142, 111–119. 10.1080/11263500701872713

[jec13160-bib-0059] Pywell, R. F. , Bullock, J. M. , Hopkins, A. , Walker, K. J. , Sparks, T. H. , Mike, J. W. B. , & Peel, S. (2002). Restoration of species‐rich grassland on arable land: Assessing the limiting processes using a multi‐site experiment. Journal of Applied Ecology, 39, 294–309. 10.1046/j.1365-2664.2002.00718.x

[jec13160-bib-0060] Pywell, R. F. , Bullock, J. M. , Tallowin, J. B. , Walker, K. J. , Warman, E. A. , & Masters, G. (2007). Enhancing diversity of species‐poor grasslands: An experimental assessment of multiple constraints. Journal of Applied Ecology, 44, 81–94. 10.1111/j.1365-2664.2006.01260.x

[jec13160-bib-0061] Quested, H. , Eriksson, O. , Fortunel, C. , & Garnier, E. (2007). Plant traits relate to whole‐community litter quality and decomposition following land use change. Functional Ecology, 21, 1016–1026. 10.1111/j.1365-2435.2007.01324.x

[jec13160-bib-0062] Reich, P. B. (2014). The world‐wide ‘fast–slow’ plant economics spectrum: A traits manifesto. Journal of Ecology, 102, 275–301. 10.1111/1365-2745.12211

[jec13160-bib-0063] Rodwell, J. S. (1998). British plant communities (Vol. 3). Cambridge, England: Cambridge University Press.

[jec13160-bib-0064] Roscher, C. , Gubsch, M. , Lipowsky, A. , Schumacher, J. , Weigelt, A. , Buchmann, N. , … Schmid, B. (2018). Trait means, trait plasticity and trait differences to other species jointly explain species performances in grasslands of varying diversity. Oikos, 127, 865–865. 10.1111/oik.04815

[jec13160-bib-0065] Roscher, C. , Schumacher, J. , Baade, J. , Wilcke, W. , Gleixner, G. , Weisser, W. W. , … Schulze, E.‐D. (2004). The role of biodiversity for element cycling and trophic interactions: An experimental approach in a grassland community. Basic and Applied Ecology, 5, 107–121. 10.1078/1439-1791-00216

[jec13160-bib-0066] Roumet, C. , Birouste, M. , Picon‐Cochard, C. , Ghestem, M. , Osman, N. , Vrignon‐Brenas, S. , … Stokes, A. (2016). Root structure‐function relationships in 74 species: Evidence of a root economics spectrum related to carbon economy. New Phytologist, 210, 815–826. 10.1111/nph.13828 26765311

[jec13160-bib-0067] Siefert, A. , Violle, C. , Chalmandrier, L. , Albert, C. H. , Taudiere, A. , Fajardo, A. , … Wardle, D. A. (2015). A global meta‐analysis of the relative extent of intraspecific trait variation in plant communities. Ecology Letters, 18, 1406–1419. 10.1111/ele.12508 26415616

[jec13160-bib-0068] Smith, R. S. , Shiel, R. S. , Bardgett, R. D. , Millward, D. , Corkhill, P. , Evans, P. , … Kometa, S. T. (2008). Long‐term change in vegetation and soil microbial communities during the phased restoration of traditional meadow grassland. Journal of Applied Ecology, 45, 670–679. 10.1111/j.1365-2664.2007.01425.x

[jec13160-bib-0069] Smith, S. W. , Woodin, S. J. , Pakeman, R. J. , Johnson, D. , & van der Wal, R. (2014). Root traits predict decomposition across a landscape‐scale grazing experiment. New Phytologist, 203, 851–862. 10.1111/nph.12845 24841886 PMC4260134

[jec13160-bib-0070] Spehn, E. M. , Hector, A. , Joshi, J. , Scherer‐Lorenzen, M. , Schmid, B. , Bazeley‐White, E. , … Lawton, J. H. (2005). Ecosystem effects of biodiversity manipulations in European grasslands. Ecological Monographs, 75, 37–63. 10.1890/03-4101

[jec13160-bib-0071] Sundqvist, M. K. , Giesler, R. , & Wardle, D. A. (2011). Within‐ and across‐species responses of plant traits and litter decomposition to elevation across contrasting vegetation types in subarctic tundra. PLoS ONE, 6, 1–12. 10.1371/journal.pone.0027056 PMC3203947 22046443

[jec13160-bib-0072] Thion, C. E. , Poirel, J. D. , Cornulier, T. , De Vries, F. T. , Bardgett, R. D. , & Prosser, J. I. (2016). Plant nitrogen‐use strategy as a driver of rhizosphere archaeal and bacterial ammonia oxidiser abundance. Fems Microbiology Ecology, 92, 11 10.1093/femsec/fiw091 27130939

[jec13160-bib-0073] Tilman, D. , Reich, P. B. , Knops, J. , Wedin, D. , Mielke, T. , & Lehman, C. (2001). Diversity and productivity in a long‐term grassland experiment. Science, 294, 843–845. 10.1126/science.1060391 11679667

[jec13160-bib-0074] Trumper, K. , Programme, U. N. E., UNEP/GRID‐Arendal & Centre, U. W. C. M. (2009). The natural fix?: The role of ecosystems in climate mitigation: A UNEP rapid response. Assessment. Cambridge, UK: United Nations Environment Programme.

[jec13160-bib-0075] Valverde‐Barrantes, O. J. , Smemo, K. A. , & Blackwood, C. B. (2015). Fine root morphology is phylogenetically structured, but nitrogen is related to the plant economics spectrum in temperate trees. Functional Ecology, 29, 796–807. 10.1111/1365-2435.12384

[jec13160-bib-0076] Villéger, S. , Mason, N. W. H. , & Mouillot, D. (2008). New multidimensional functional diversity indices for a multifaceted framework in functional ecology. Ecology, 89, 2290–2301. 10.1890/07-1206.1 18724739

[jec13160-bib-0077] Ward, S. E. , Bardgett, R. D. , McNamara, N. P. , Adamson, J. K. , & Ostle, N. J. (2007). Long‐term consequences of grazing and burning on northern peatland carbon dynamics. Ecosystems, 10, 1069–1083. 10.1007/s10021-007-9080-5

[jec13160-bib-0078] Wardle, D. A. (2016). Do experiments exploring plant diversity–ecosystem functioning relationships inform how biodiversity loss impacts natural ecosystems? Journal of Vegetation Science, 27, 646–653.

[jec13160-bib-0079] Weemstra, M. , Mommer, L. , Visser, E. J. W. , van Ruijven, J. , Kuyper, T. W. , Mohren, G. M. J. , & Sterck, F. J. (2016). Towards a multidimensional root trait framework: A tree root review. New Phytologist, 211, 1159–1169. 10.1111/nph.14003 27174359

[jec13160-bib-0080] White, D. C. , Davis, W. M. , Nickels, J. S. , King, J. D. , & Bobbie, R. J. (1979). Determination of the sedimentary microbial biomass by extractible lipid phosphate. Oecologia, 40, 51–62. 10.1007/BF00388810 28309603

[jec13160-bib-0081] Wright, I. J. , Reich, P. B. , Westoby, M. , Ackerly, D. D. , Baruch, Z. , Bongers, F. , … Villar, R. (2004). The worldwide leaf economics spectrum. Nature, 428, 821–827. 10.1038/nature02403 15103368

[jec13160-bib-0082] Zemunik, G. , Turner, B. L. , Lambers, H. , & Laliberté, E. (2015). Diversity of plant nutrient‐acquisition strategies increases during long‐term ecosystem development. Nature Plants, 1, 15050 10.1038/nplants.2015.50

[jec13160-bib-0083] Zirbel, C. R. , Bassett, T. , Grman, E. , & Brudvig, L. A. (2017). Plant functional traits and environmental conditions shape community assembly and ecosystem functioning during restoration. Journal of Applied Ecology, 54, 1070–1079. 10.1111/1365-2664.12885

[jec13160-bib-0084] Zuo, X. , Zhou, X. , Lv, P. , Zhao, X. , Zhang, J. , Wang, S. , & Yue, X. (2016). Testing associations of plant functional diversity with carbon and nitrogen storage along a restoration gradient of sandy grassland. Frontiers in Plant Science, 7, 1–11. 10.3389/fpls.2016.00189 26925089 PMC4759253

