# Supplementary material for: Relationships between plant traits, soil properties and carbon fluxes differ between monocultures and mixed communities in temperate grassland
Source: J Ecol. 2019 Mar 25;107(4):1704–19. doi: 10.1111/1365-2745.13160 (PMC6617750; doi:10.1111/1365-2745.13160)
Supplement: Supplementary file 1 [file JEC-107-1704-s001.docx]

**Supporting Information**

“Relationships between plant traits, soil properties and carbon fluxes differ between monocultures and mixed communities in temperate grassland.”

Jonathan R. De Long^*^, Benjamin G. Jackson^*^, Anna Wilkinson, William J. Pritchard, Simon Oakley, Kelly E. Mason, Jörg G. Stephan, Nicholas J. Ostle, David Johnson, Elizabeth M. Baggs and Richard D. Bardgett

**Contents**

**Table S1.** Mean trait values of plant species grown in the monoculture experiment.

**Table S2.** Species planted and sown into community experiment.

**Table S3.** Sowing rates for the community experiment.

**Table S4.** Numbers of seedlings planted in the community experiment.

**Table S5.** Relative abundance of plant species used to characterise CWM shoot traits in the community experiment.

**Figure S1.** Monoculture species coverage in community experiment.

**Figure S2.** Principle component analyses of above and below ground trait covariation in the monoculture and community experiments.

**Figure S3.** Principle component analyses of covariation of soil properties in the monoculture experiment.

**Figure S4.** Principle component analyses of covariation of soil properties in the community experiment.

**Table S6.** Results of standard major axis (SMA) regressions.

**Figure S5.** Relationships between principle components of shoot and root trait covariation and soil properties in the monoculture and community experiments.

**Methods S1.** Path models: construction, model simplification and validation.

**Table S7.** Regression matrix of relationships between shoot and root traits from monoculture experiment.

**Table S8.** Regression matrix of relationships between plant traits and selected soil properties from the monoculture experiment.

**Figure S6.** Percentage cover and species richness of the target functional groups in the mixed community experiment.

**Table S9.** Regression matrix of relationships between community weighted mean (CWM) shoot traits and root traits in the community experiment.

**Table S10.** Regression matrix of relationships between plant traits and soil abiotic and biotic properties in the mixed community experiment.

**Figure S7.** Effect of plant species addition treatments on community weighted mean (CWM) shoot nitrogen (N) content in the mixed community experiment.

**Table S11.** Summary statistics for functional traits, soil abiotic properties and the soil microbial community from the monoculture experiment.

**Table S12**. Summary statistics for functional traits, soil abiotic properties and the soil microbial community from the mixed community experiment.

Table S1. Functional group and mean values (±SD) for selected traits of the 25 plant species grown the monoculture experiment. Absence of standard deviations indicate single record for the trait and/or species.

| Species | Functional  Group | SLA  (cm^2^ g^-1^) | | | LDMC  (mg g^-1^) | | | Shoot N  (%) | | | SRL  (m g^-1^) | | | RDMC  (mg g^-1^) | | | Root N  (%) | | |
| --- | --- | --- | --- | --- | --- | --- | --- | --- | --- | --- | --- | --- | --- | --- | --- | --- | --- | --- | --- |
| *Agrostis capillaris* | Grass | 27.05 | ± | 3.47 | 264.15 | ± | 6.70 | 2.73 | ± | 0.13 | 196.73 | ± | 56.13 | 161.98 | ± | 14.46 | 0.71 | ± | 0.04 |
| *Anthoxanthum odoratum* | Grass | 26.51 | ± | 2.47 | 231.26 | ± | 17.18 | 2.77 | ± | 0.37 | 290.36 | ± | 12.92 | 167.05 | ± | 24.98 | 0.65 | ± | 0.09 |
| *Briza media* | Grass | 25.10 | ± | 2.17 | 280.36 | ± | 15.99 | 2.13 | ± | 0.27 | 185.40 | ± | 34.27 | 151.51 | ± | 7.08 | 0.57 | ± | 0.05 |
| *Cynosurus cristatus* | Grass | 28.93 | ± | 2.02 | 217.67 | ± | 14.12 | 2.99 | ± | 0.69 | 389.35 | ± | 81.43 | 123.88 | ± | 17.73 | 0.98 | ± | 0.22 |
| *Dactylis glomerata* | Grass | 33.36 | ± | 3.03 | 229.80 | ± | 24.16 | 3.00 | ± | 1.00 | 187.50 | ± | 94.67 | 232.64 | ± | 65.54 | 0.71 | ± | 0.15 |
| *Festuca rubra* | Grass | 12.20 | ± | 1.71 | 301.95 | ± | 25.04 | 2.20 | ± | 0.49 | 160.75 | ± | 104.92 | 160.36 | ± | 7.65 | 0.80 | ± | 0.10 |
| *Holcus lanatus* | Grass | 45.61 |  |  | 194.17 |  |  | 4.10 |  |  | 335.16 |  |  | 171.78 |  |  | 1.02 |  |  |
| *Achillea millefolium* | Forb | 16.65 | ± | 2.04 | 165.97 | ± | 5.03 | 3.00 | ± | 0.21 | 46.86 | ± | 21.10 | 133.66 | ± | 20.66 | 0.81 | ± | 0.06 |
| *Bellis perennis* | Forb | 37.48 | ± | 2.70 | 122.27 | ± | 8.84 | 2.75 | ± | 0.36 | 127.11 | ± | 51.25 | 116.97 | ± | 18.97 | 1.25 | ± | 0.19 |
| *Centaurea nigra* | Forb | 21.64 | ± | 1.20 | 155.09 | ± | 4.91 | 2.23 | ± | 0.31 | 91.33 | ± | 20.09 | 138.02 | ± | 15.32 | 0.74 | ± | 0.11 |
| *Filipendula ulmaria* | Forb | 22.20 | ± | 1.91 | 280.89 | ± | 9.68 | 2.88 | ± | 0.74 | 66.06 | ± | 28.48 | 202.54 | ± | 36.15 | 0.74 | ± | 0.08 |
| *Geranium pratense* | Forb | 23.56 | ± | 3.02 | 247.64 | ± | 12.53 | 2.02 | ± | 0.35 | 20.01 | ± | 5.73 | 221.11 | ± | 50.26 | 0.37 | ± | 0.04 |
| *Geranium sylvaticum* | Forb | 24.17 | ± | 1.57 | 244.12 | ± | 9.17 | 2.20 | ± | 0.14 | 35.44 | ± | 11.92 | 243.58 | ± | 18.04 | 0.62 | ± | 0.10 |
| *Geum rivale* | Forb | 17.36 | ± | 1.14 | 294.74 | ± | 76.58 | 1.82 | ± | 0.08 | 118.09 | ± | 47.28 | 169.31 | ± | 9.94 | 0.87 | ± | 0.17 |
| *Hypochaeris radicata* | Forb | 13.75 | ± | 1.76 | 181.06 | ± | 7.14 | 0.78 | ± | 0.15 | 242.13 | ± | 79.11 | 86.88 | ± | 14.84 | 0.97 | ± | 0.25 |
| *Leontodon hispidus* | Forb | 25.01 | ± | 2.72 | 135.92 | ± | 10.68 | 2.73 | ± | 0.18 | 223.15 | ± | 77.22 | 98.73 | ± | 9.56 | 1.05 | ± | 0.10 |
| *Leucanthemum vulgare* | Forb | 17.55 | ± | 3.14 | 168.94 | ± | 17.48 | 1.39 | ± | 0.32 | 100.36 | ± | 43.15 | 138.70 | ± | 23.91 | 0.69 | ± | 0.14 |
| *Plantago lanceolata* | Forb | 17.93 | ± | 1.67 | 147.00 | ± | 4.84 | 2.53 | ± | 0.51 | 115.67 | ± | 32.07 | 172.99 | ± | 39.57 | 1.05 | ± | 0.12 |
| *Ranunculus acris* | Forb | 26.98 |  |  | 158.33 |  |  | 2.49 |  |  | 68.56 |  |  | 173.22 |  |  | 0.65 |  |  |
| *Ranunculus repens* | Forb | 19.47 |  |  | 183.86 |  |  | 1.64 |  |  | 45.38 | ± | 37.13 | 180.32 | ± | 30.48 | 0.79 | ± | 0.19 |
| *Rumex acetosa* | Forb | 30.17 | ± | 4.03 | 114.90 | ± | 10.30 | 3.71 | ± | 0.21 | 228.15 | ± | 135.07 | 191.97 | ± | 33.39 | 1.18 | ± | 0.31 |
| *Lathyrus pratensis* | Legume | 27.35 | ± | 6.54 | 226.92 | ± | 36.65 | 4.47 | ± | 0.70 | 16.95 | ± | 5.53 | 190.57 | ± | 18.78 | 2.66 | ± | 0.04 |
| *Lotus corniculatus* | Legume | 25.79 | ± | 1.37 | 192.05 | ± | 7.16 | 3.90 | ± | 0.20 | 52.87 | ± | 24.15 | 166.09 | ± | 41.04 | 2.06 | ± | 0.11 |
| *Trifolium pratense* | Legume | 30.24 | ± | 1.73 | 206.56 | ± | 2.07 | 4.36 | ± | 0.24 | 94.63 | ± | 25.86 | 148.89 | ± | 17.66 | 2.15 | ± | 0.21 |
| *Trifolium repens* | Legume | 22.82 |  |  | 201.49 |  |  | 3.84 |  |  | 243.33 |  |  | 134.69 |  |  | 2.27 |  |  |

Table S2. Plant species from different functional groups (i.e., grasses, forbs, legumes) were added to treatment plots as seeds and seedlings during the course of the experiment.

|  |  |  |  | Seeds |  |  | Seedlings | |  |
| --- | --- | --- | --- | --- | --- | --- | --- | --- | --- |
| Species |  | Functional group |  | 2014^*^ | 2015^+^ |  | 2013^**^ | 2014^*^ | 2015^*^ |
| *Cynosurus cristatus* |  | Grass |  | X | X |  | X | X |  |
| *Dactylis glomerata* |  | Grass |  | X | X |  | X | X | X |
| *Festuca rubra* |  | Grass |  | X | X |  | X | X |  |
| *Poa trivialis* |  | Grass |  | X |  |  | X | X |  |
| *Briza media* |  | Grass |  | X | X |  | X | X | X |
| *Achillea millefolium* |  | Forb |  | X | X |  | X | X | X |
| *Geranium sylvaticum* |  | Forb |  | X | X |  | X | X | X |
| *Geum rivale* |  | Forb |  | X | X |  | X | X | X |
| *Leucanthemum vulgare* |  | Forb |  | X | X |  | X | X | X |
| *Plantago lanceolata* |  | Forb |  | X |  |  | X | X |  |
| *Prunella vulgaris* |  | Forb |  | X | X |  | X | X |  |
| *Hypochaeris radicata* |  | Forb |  | X | X |  | X | X |  |
| *Leontodon hispidus* |  | Forb |  | X | X |  | X | X |  |
| *Filipendula ulmaria* |  | Forb |  | X | X |  | X | X | X |
| *Centaurea nigra* |  | Forb |  | X |  |  | X | X |  |
| *Lathyrus pratensis* |  | Legume |  | X | X |  | X | X | X |
| *Lotus corniculatus* |  | Legume |  | X | X |  | X | X | X |
| *Trifolium pratense* |  | Legume |  | X | X |  | X | X |  |
| *Trifolium repens* |  | Legume |  | X |  |  | X | X |  |

^*^ Seeded in May

^**^ Planted in May and September

^+^ Seeded in March, April, and September

Table S3. Number of seeds from each functional group sown per plot in the different PFG treatments. See Table S1 for frequency of sowing and which species from each functional group were used.

| 2014 |  |  |  |  |  |  |  |  |  |
| --- | --- | --- | --- | --- | --- | --- | --- | --- | --- |
|  |  | Functional group | |  |  |  |  |  |  |
| Treatment |  | Total forbs | Each forb species |  | Total legumes | Each legume species |  | Total grasses | Each grass species |
| Control |  | 0 | 0 |  | 0 | 0 |  | 0 | 0 |
| Forbs (F) |  | 64800 | 8100 |  | 0 | 0 |  | 0 | 0 |
| Legumes (L) |  | 0 | 0 |  | 64800 | 21600 |  | 0 | 0 |
| Grasses (G) |  | 0 | 0 |  | 0 | 0 |  | 64800 | 16200 |
| G+F |  | 32400 | 4050 |  | 0 | 0 |  | 32400 | 8100 |
| G+L |  | 0 | 0 |  | 32400 | 10800 |  | 32400 | 8100 |
| F+L |  | 32400 | 4050 |  | 32400 | 10800 |  | 0 | 0 |
| G+F+L |  | 21600 | 2700 |  | 21600 | 7200 |  | 21600 | 5400 |
| Total |  | 151200 | 18900 |  | 151200 | 50400 |  | 151200 | 37800 |

| 2015 |  |  |  |  |  |  |  |  |  |
| --- | --- | --- | --- | --- | --- | --- | --- | --- | --- |
|  |  | Functional group | |  |  |  |  |  |  |
| Treatment |  | Total forbs | Each forb species |  | Total legumes | Each legume species |  | Total grasses | Each grass species |
| Control |  | 0 | 0 |  | 0 | 0 |  | 0 | 0 |
| Forbs (F) |  | 97200 | 12150 |  | 0 | 0 |  | 0 | 0 |
| Legumes (L) |  | 0 | 0 |  | 97200 | 32400 |  | 0 | 0 |
| Grasses (G) |  | 0 | 0 |  | 0 | 0 |  | 97200 | 24300 |
| G+F |  | 48600 | 6075 |  | 0 | 0 |  | 48600 | 12150 |
| G+L |  | 0 | 0 |  | 48600 | 16200 |  | 48600 | 12150 |
| F+L |  | 48600 | 6075 |  | 48600 | 16200 |  | 0 | 0 |
| G+F+L |  | 32400 | 4050 |  | 32400 | 10800 |  | 32400 | 8100 |
| Total |  | 226800 | 28350 |  | 226800 | 75600 |  | 226800 | 56700 |

Table S4. Number of plugs of different species from each functional group sown per 6 x 6 m plot from the different plant functional group treatments. See Table S1 for species used and in which year each species was planted. Within each functional group, an equal number of each species was planted.

| 2013, 2014 |  |  |  |  |
| --- | --- | --- | --- | --- |
|  |  | Functional group | |  |
| Treatment |  | Forbs | Legumes | Grasses |
| Control |  | 0 | 0 | 0 |
| Forbs (F) |  | 300 | 0 | 0 |
| Legumes (L) |  | 0 | 300 | 0 |
| Grasses (G) |  | 0 | 0 | 300 |
| G+F |  | 150 | 0 | 150 |
| G+L |  | 0 | 150 | 150 |
| F+L |  | 150 | 150 | 0 |
| G+F+L |  | 100 | 100 | 100 |
| Total |  | 3500 | 3500 | 3500 |

| 2015 |  |  |  |  |
| --- | --- | --- | --- | --- |
|  |  | Functional group | |  |
| Treatment |  | Forbs | Legumes | Grasses |
| Control |  | 0 | 0 | 0 |
| Forbs (F) |  | 100 | 0 | 0 |
| Legumes (L) |  | 0 | 100 | 0 |
| Grasses (G) |  | 0 | 0 | 100 |
| G+F |  | 50 | 0 | 50 |
| G+L |  | 0 | 50 | 50 |
| F+L |  | 50 | 50 | 0 |
| G+F+L |  | 33 | 33 | 33 |
| Total |  | 1150 | 1150 | 1150 |

Table S5. Species collected from the control and grass, forb and legume addition plots in the field communities to perform measurements on leaf traits. Numbers shown are mean ± SE; n = 5 for each treatment.

|  |  |  |  |  |
| --- | --- | --- | --- | --- |
|  |  | Average % cover (corrected) | |  |
| Treatment |  | Control | Grass, forb, legume addition | Functional group |
| *Agrostis capillaris* |  | 16.00 ± 2.61 | 15.80 ± 1.43 | grass |
| *Anthoxanthum odoratum* |  | 19.40 ± 3.08 | 21.00 ± 3.38 | grass |
| *Bellis perennis* |  | 6.60 ± 1.44 | 8.40 ± 2.20 | forb |
| *Festuca rubra* |  | 3.20 ± 0.97 | 2.90 ± 0.40 | grass |
| *Holcus lanatus* |  | 15.20 ± 1.88 | 16.00 ± 2.70 | grass |
| *Lathyrus pratensis* |  | 0.00 ± 0.00 | 1.60 ± 0.19 | legume |
| *Lotus corniculatus* |  | 0.00 ± 0.00 | 1.00 ± 0.00 | legume |
| *Plantago lanceolata* |  | 0.40 ± 0.24 | 1.40 ± 0.19 | forb |
| *Poa trivialis* |  | 3.00 ± 1.00 | 4.40 ± 1.61 | grass |
| *Ranunculus acris* |  | 17.80 ± 1.85 | 18.20 ± 1.93 | forb |
| *Ranunculus repens* |  | 15.80 ± 1.69 | 12.40 ± 2.16 | forb |
| *Rhinanthus minor* |  | 2.20 ± 0.25 | 3.50 ± 1.02 | hemi-parasite |
| *Rumex acetosa* |  | 7.00 ± 0.55 | 7.60 ± 0.81 | forb |
| *Trifolium pratense* |  | 0.80 ± 0.37 | 1.80 ± 0.46 | legume |
| *Trifolium repens* |  | 1.90 ± 0.29 | 2.60 ± 0.43 | legume |
| Total average % cover captured |  | **88.89 ± 2.61** | **81.24 ± 2.90** |  |

Fig. S1. Proportion of total vegetation cover in the field community plots represented by plant species included in the monoculture experiment. Data show mean cover per species (n=5) with functional group addition treatments across the x-axis (C = control, F = forb addition, L = legume addition, G = grass addition).

Fig. S2. Principle component analyses (PCA) showing the patterns of covariation in leaf and root traits for 25 temperate grassland species grown in monocultures (a^†^, b^†^) and for 40 grassland communities (c^‡^, d^‡^) resulting from experimental manipulation of the diversity and functional composition of plant species in the swards.

† Functional groups are shown as red = grasses, blue = forbs and green = legumes. Species names in panels (a) and (b) are ACHMIL = *Achillea millefolium*, AGRCAP = *Agrostis capillaris*, ANTODO = *Anthoxanthum odoratum*, BELPER = *Bellis perennis*, BRIMED = *Briza media*, CENNIG = *Centaurea nigra*, CYNCRI = *Cynosurus cristatus*, DACGLO = *Dactylis glomerata*, FESRUB = *Festuca rubra*, FILULM = *Filipendula ulmaria*, GERPRA = *Geranium pratense*, GERSYL = *Geranium sylvaticum*, GEURIV = *Geum rivale*, HOLLAN = *Holcus lanatus*, HYPRAD = *Hypochaeris radicata*, LATPRA = *Lathyrus pratensis*, LEOHIS = *Leontodon hispidus*, LEUVUL = *Leucanthemum vulgare*, LOTCOR = *Lotus corniculatus*, PLALAN = *Plantago lanceolata*, RANACR = *Ranunculus acris*, RANREP = *Ranunculus repens*, RUMACE = *Rumex acetosa*, TRIPRA = *Trifolium pratense*, TRIREP = *Trifolium repens*.

‡ Functional diversity manipulations in panels (c) and (d) are shown as black = control, red = grasses, blue = forbs, green = legumes, purple = grasses + forbs, orange = grasses + legumes, yellow = forbs + legumes, grey = grasses + forbs + legumes.

Fig. S3. Principal component analysis (PCA) using selected soil properties from monoculture soils in which 25 grassland plant species were grown. Functional groups are colour coded (grasses = red, forbs = blue, legumes = green). PLFA = phospholipid fatty acid.

ACHMIL = *Achillea millefolium*, AGRCAP = *Agrostis capillaris*, ANTODO = *Anthoxanthum odoratum*, BELPER = *Bellis perennis*, BRIMED = *Briza media*, CENNIG = *Centaurea nigra*, CYNCRI = *Cynosurus cristatus*, DACGLO = *Dactylis glomerata*, FESRUB = *Festuca rubra*, FILULM = *Filipendula ulmaria*, GERPRA = *Geranium pratense*, GERSYL = *Geranium sylvaticum*, GEURIV = *Geum rivale*, HOLLAN = *Holcus lanatus*, HYPRAD = *Hypochaeris radicata*, LATPRA = *Lathyrus pratensis*, LEOHIS = *Leontodon hispidus*, LEUVUL = *Leucanthemum vulgare*, LOTCOR = *Lotus corniculatus*, PLALAN = *Plantago lanceolata*, RANACR = *Ranunculus acris*, RANREP = *Ranunculus repens*, RUMACE = *Rumex acetosa*, TRIPRA = *Trifolium pratense*, TRIREP = *Trifolium repens*.

Fig. S4. Principal component analysis (PCA) using selected soil properties from 40 plots subjected to plant functional diversity manipulation. Treatments are shown as black = control, red = grasses, blue = forbs, green = legumes, purple = grasses + forbs, orange = grasses + legumes, yellow = forbs + legumes, grey = grasses + forbs + legumes. PLFA = phospholipid fatty acid.

Table S6. Results of standard major axis (SMA) regressions (shown in Fig. S5) between principle axes of variation from the PCAs of shoot and root traits (Fig. S2) and soil properties (Figs. S3 and S4) for the monoculture and community experiments, respectively. Data show *R*^2^ (*P* values) from model II regressions.

|  |  | Monoculture  Experiment | Community  Experiment |
| --- | --- | --- | --- |
| Shoot traits *vs.* root traits | Shoot PC1 vs Root PC1 | **0.19 (<0.001)** | **0.10 (0.047)** |
|  | Shoot PC1 vs Root PC2 | 0.01 (0.318) | 0.01 (0.667) |
|  | Shoot PC2 vs Root PC1 | **0.11 (0.005)** | 0.03 (0.322) |
|  | Shoot PC2 vs Root PC2 | **0.06 (0.034)** | 0.01 (0.538) |
| Shoot traits *vs.* soil properties | Shoot PC1 vs Soil PC1 | 0.01 (0.414) | 0.04 (0.251) |
|  | Shoot PC1 vs Soil PC2 | **0.29 (<0.001)** | 0.00 (0.971) |
|  | Shoot PC1 vs Soil PC3 | 0.00 (0.869) | 0.03 (0.304) |
|  | Shoot PC2 vs Soil PC1 | 0.00 (0.843) | 0.07 (0.129) |
|  | Shoot PC2 vs Soil PC2 | 0.02 (0.264) | 0.02 (0.411) |
|  | Shoot PC2 vs Soil PC3 | 0.00 (0.605) | 0.02 (0.417) |
| Root traits *vs.* soil properties | Root PC1 vs Soil PC1 | 0.00 (0.732) | **0.12 (0.042)** |
|  | Root PC1 vs Soil PC2 | **0.08 (0.016)** | 0.02 (0.405) |
|  | Root PC1 vs Soil PC3 | 0.00 (0.768) | 0.01 (0.679) |
|  | Root PC2 vs Soil PC1 | 0.00 (0.995) | 0.01 (0.637) |
|  | Root PC2 vs Soil PC2 | 0.00 (0.871) | 0.01 (0.529) |
|  | Root PC2 vs Soil PC3 | 0.00 (0.940) | 0.00 (0.792) |

Figure S5. Standardized major axes regressions showing the relationships between principle components (PC) of variation in leaf traits, root traits and soil properties in monocultures (a, c and e) and community plots (b, d, and f). Principal components are derived from the ordinations in Figs. 1 and S7. Labeled arrows indicate scaling of representative variables along each PC. In the monocultures (panels a, c and e) functional groups are shown as red = grasses, blue = forbs and green = legumes. In the community plots (panels b, d and f) functional diversity manipulations are shown as black = control, red = grasses, blue = forbs, green = legumes, purple = grasses + forbs, orange = grasses + legumes, yellow = forbs + legumes, grey = grasses + forbs + legumes.

Methods S1. Path models: construction, model simplification and validation

For the path analyses of the monoculture and community experiments, we used partial least square path models (PLS-PMs.). This type of structural equation modelling (SEM) is becoming more prominent in natural science (Majdi *et al*. 2014; Gorai *et al*. 2015; Musseau *et al*. 2015; Stephan *et al*. 2017) and has no distributional or independence requirements for the used data (Fornell & Bookstein 1982; Chin & Dibbern 2010). The relationship between latent variables (LVs) with several indicators can be estimated even when sample size is small (Chin 1999). This is mainly due to the reliance on ordinal least squares for each component of the model; meaning model complexity is relatively independent of replication number. In cases were covariance-based SEMs (CB-SEM) do not converge, this method is more robust and therefore favourable, although non-convergence may occur also (Henseler 2010). The main difference between the two approaches is that CB-SEM focuses on aligning the sample covariance matrix to the theoretical covariance matrix, while PLS-PM aims to maximize the explained variance of the latent variables (Reinartz *et al*. 2009). While the respective performance of the two approaches can differ in certain circumstances, both approaches generally provide comparable results (Chin & Dibbern 2010). We chose PLS-PM also because we had many measured variables that can be included in LVs. PLS-PM performs very well when using LVs and therefore also avoids inflating model complexity with unnecessarily large numbers of potential paths. The models were run with the reflective mode, with each (dependent) indicator being regressed against the (independent) LV score in an iterative process (for a description on the algorithm of PLS-PM see e.g. (Henseler 2010)). The outer model (measured variables forming LVs; called measurement model within CB-SEM) and inner model (LVs and paths; called structural model within CB-SEM) of all six PLS-PMs were built as follows:

*Model specification*

Informed by the exploratory analyses of the data from the two experiments, in particular the ordinations shown in Figures 1, and Figures S3 and S4, subsets of the measured variables from each experiment were selected to be used as reflective indicators of latent variables. Latent variables were defined to represent the broader patterns of biotic and abiotic variation observed in our system; see Methods S1, Table A for the complete list of variables included in each outer model. Then, based on (i) prior understanding of the study system, (ii) previously reported trait–soil–process linkages (see Fig. S2), (iii) the experimental designs employed and (iv) our interest in testing weather relationships between plant traits, soil properties and ecosystem carbon fluxes (i.e., net ecosystem exchange, ecosystem respiration) scale from the individual species to mixed plant communities, a common *a priori* model structure was constructed.

*Model Evaluation*

For the outer model, we used the factorial weighting scheme, which ignores the directionality of the paths among latent variables (Chin & Dibbern 2010). In reflective mode, the importance of the indicators needs to be inspected based on loadings (i.e., the contribution of each measured indicator to the path coefficient, with values higher than 0.7 indicating a strong effect) and cross loadings (i.e., the contribution of each measured indicator to other LVs, which need to be highest for its own LV). Before checking the loadings, we made sure that each indicator fulfilled the requirement of unidimensionality (i.e., all loadings were positive) and multiplied the indicator with minus one if this was violated (this procedure was repeated after any alteration of the model). This unidimensionality is also indicated by the composite reliability (Dillon-Goldstein’s rho) of each LV, which was always larger than the recommended 0.7 for all final models (Vinzi *et al*. 2010). We then evaluated the outer model by removing indicators that had loadings lower than 0.6, which is restrictive. The evaluation of how well a LV is described by its indicators can be done using the average variance extracted (AVE) that describes the variance captured from indicators compared to variance due to measurement error. After removal of less important indicators, all AVEs were larger than the recommended 0.5 (the minimum was 0.66). Furthermore, all indicators had the highest cross loading for their respective LV. Recently, the Goodness-of-Fit (GoF) has been shown to be less suitable to evaluate the overall model, especially the inner model. However, it can be utilized to judge the outer model (Henseler & Sarstedt 2013). All GoFs were above 0.41 (0.37 for the functional group models), which is acceptable (values of 0.7 are considered highly acceptable (Hair *et al*. 2011)). To evaluate the paths in the inner model, we specified the path-weighting scheme (accounts for strength and direction of the paths in the inner model (Vinzi *et al*. 2010)) on the previously derived model. As recommended (Henseler & Sarstedt 2013), we only considered significant paths in a model. All other paths were removed from the model. As a result LVs (and their respective measured indicators) that ended up with no path directed from or to them were removed entirely from the model.

*Model validation*

Several steps were undertaken to validate the final model. The loadings and the path coefficients were bootstrapped (100 resamplings) and revealed no differences to the original values (results shown in Methods S1, Tables B-G below). To validate model convergence, we estimated all final models with all three weighting schemes (centroid, path, factorial) (Henseler 2010), which showed only marginal difference (e.g., Coltpart-NEE: AVE for Soil properties changed from 0.703 to 0.705). Lastly, we used Stone-Geisser’s predictive relevance (Q^2^) as information theoretical model evaluation criteria. Q^2^ can be used for reflective indicators on endogenous LVs and is the most reliable index that balances model fit and parsimony (Sharma & Kim 2012). It omits part of the data for an indicator and then attempts to estimate the omitted part using the previously estimated parameters. For this iterative blindfolding process, we used the minimum omission distance of five data points (Hair *et al*. 2017). Larger than zero values indicate predictive relevance (Chin & Dibbern 2010), which was the case for all Q^2^ values of all final models. Model specification and evaluation was performed using R (R Core Team, 2016) and the package *plspm* (Sanchez *et al*. 2015). However, Q^2^ could only be calculated using the package *semPLS* (Monecke & Leisch 2012). All final models were therefore also re-coded for the *semPLS* package, and this could be seen as a technical model validation as we observed only differences in the predictors/indices due to rounding.

*Interpreting the model*

Within the model, the inner model represents a causal chain and the outer model (in reflective mode) assumes a causal relationship from the LVs to the indicators (Henseler 2010). Therefore, direction of effects can be summarized. For example, SLA has a negative effect on shoot dry weight (-0.41) and shoot dry weight has a negative effect on net ecosystem exchange (-59; Fig. 3a), meaning SLA has an overall positive indirect effect on net ecosystem exchange. This is also represented by the indirect effect of leaf traits on net ecosystem exchange (0.40), however, this effect arises from all possible direct connections (in this case also: leaf traits -> root nutrients -> microbial community -> soil properties -> net ecosystem exchange; leaf traits -> root nutrients -> root biomass -> shoot dry weight -> net ecosystem exchange).

*References*

Chin, W. W. (1999) Structural Equation Modelling Analysis With Small Samples Using Partial Least Squares. In: Hoyle, R. H. (Ed.), *Statistical strategies for small sample research (*pp. 307–341). London, England: SAGE Publications Inc.,

Chin, W. W. & Dibbern, J. (2010) How to Write Up and Report PLS Analyses. In: Vinzi, V. E., Chin, W. W., Henseler, J., & Wang, H (Eds.), *Handbook of Partial Least Squares Concepts, Methods and Applications* (pp. 171–193)*.* Heidelberg: Springer.

Fornell, C. & Bookstein, F. L. (1982) Two Structural Equation Models: LISREL and PLS Applied to Consumer Exit-Voice Theory. *Journal of Marketing Research,* **19**, 440-452.

Gorai, A. K., Tuluri, F. & Tchounwou, P. B. (2015) Development of PLS-path model for understanding the precursors of ground level ozone concentration in Gulfport, Mississippi, USA. *Atmospheric Pollution Research,* **6**, 389–397.

Hair, J. F., Ringle, C. M. & Sarstedt, M. (2011) PLS-SEM: Indeed a Silver Bullet. *Journal of Marketing Theory and Practice,* **19**, 139–152.

Hair, J. F., Hult, G. T. M., Ringle, C. & Sarstedt M. (2017) A primer on partial least squares structural equation modelling (PLS-SEM). London, England: SAGE.

Henseler, J. (2010) On the convergence of the partial least squares path modelling algorithm. *Computational Statistics,* **25**, 107–120.

Henseler, J. & Sarstedt, M. (2013) Goodness-of-fit indices for partial least squares path modelling. *Computational Statistics,* **28**, 565–580.

Majdi, N., Boiché, A., Traunspurger, W. & Lecerf, A. (2014) Predator effects on a detritus-based food web are primarily mediated by non-trophic interactions. *Journal of Animal Ecology,* **83**, 953–962.

Monecke, A. & Leisch, F. (2012) SemPLS: structural equation modelling using partial least squares. *Journal of Statistical Software*, **48**, doi: 10.18637/jss.v048.i03

Musseau, C., Vincenzi, S., Jesensek, D., Canter, I., Boulêtreau, S., Santoul, F. & Crivelli, A. J. (2015) Direct and indirect effects of environmental factors on dietary niches in size-structured populations of a wild salmonid. *Ecosphere,* **6**, 1–15.

Reinartz, W., Haenlein, M. & Henseler, J. (2009) An empirical comparison of the efficacy of covariance-based and variance- based SEM. *International Journal of Marketing Research,* **26**, 332–344.

R Core Team. (2016) R: A language and environment for statistical computing. R Foundation for Statistical Computing, Vienna, Austria. URL http://www.R-project.org/.

Sanchez, G., Trinchera, L. & Russolillo, G. (2015) Tools for Partial Least Squares Path Modelling (PLS-PM). https://cran.r-project.org/web/packages/plspm/index.html

Sharma, P. & Kim, K. (2012) Model Selection in Information Systems Research Using Partial Least Squares Based Structural Equation Modelling. *Icis*, 1–13.

Stephan, J. G., Pourazari, F., Tattersdill, K., Kobayashi, T., Nishizawa, K. & De Long, J. R. (2017) Long-term deer exclosure alters soil properties, plant traits, understory plant community and insect herbivory, but not the functional relationships among them. *Oecologia,* **184**, 685–699.

Vinzi V. E., Trinchera L. & Amato S. (2010) PLS Path Modelling: From Foundations to Recent Developments and Open Issues for Model Assessment and Improvement. In: Vinzi V. E., Chin W., Henseler J., Wang H. (Eds.), Handbook of Partial Least Squares. Springer Handbooks of Computational Statistics (pp. 171-193). Heidelberg: Springer.

Methods S1, Table A. Measured variables used as reflective indicators of latent variables in the starting models for each of the path analyses of the monoculture and community experiments.

| Latent variable | Monoculture experiment | Community experiment |
| --- | --- | --- |
| Leaf traits | Shoot C  Shoot N  Shoot C:N  LDMC  SLA | Shoot C  Shoot N  Shoot C:N  LDMC  SLA |
| Root nutrients | Root C  Root N  Root C:N  RDMC | Root C  Root N  Root C:N |
| Root morphology | SRL  Root diameter | SRL  Root Diameter |
| Soil properties | NO_3_-N  NH_4_-N  Soil C  Soil N  pH | NO_3_-N  NH_4_-N  Soil C  Soil N  pH |
| Microbial community | Microbial biomass C:N  Total fungal PLFA  Total bacterial PLFA  Fungal to bacterial ratio (PLFA)  Gram pos. bacteria (PLFA)  Gram neg. bacteria (PLFA)  Gram pos to Gram neg ratio | Microbial biomass C:N  Total fungal PLFA  Total bacterial PLFA  Gram pos. bacteria (PLFA)  Gram neg. bacteria (PLFA) |
| Root biomass allocation | Root dry weight per unit soil volume  Root length per unit soil volume  Root surface area per unit soil volume | Root dry weight per metre square  Root to shoot ratio |
| Shoot biomass | Shoot biomass per pot | Shoot biomass per metre square |
| Ecosystem function | Mean growing season net ecosystem exchange and ecosystem respiration (4 dates) | Mean growing season net ecosystem exchange and ecosystem respiration (9 dates) |

C = carbon, LDMC = leaf dry matter content, N = nitrogen, SLA = specific leaf area, SRL = specific root length.

Methods S1, Table B. Original and bootstrapped loadings for each measured indicator for all latent variables from the final model on predicting net ecosystem exchange in the monoculture experiment (shown in Figure 3a).

| Latent variable | Measured indicator | Original | Mean | SE |
| --- | --- | --- | --- | --- |
| Leaf traits | Shoot N | 0.95 | 0.95 | 0.01 |
| Leaf traits | SLA | 0.77 | 0.76 | 0.06 |
| Leaf traits | C:N ratio | 0.90 | 0.91 | 0.02 |
| Root morphology | SRL | 1.00 | 1.00 | 0.00 |
| Root nutrients | Root N | 0.96 | 0.96 | 0.01 |
| Root nutrients | C:N ratio | 0.95 | 0.95 | 0.01 |
| Soil microbial community | Fungal to bacterial ratio | 0.77 | 0.76 | 0.02 |
| Soil microbial community | Gram- PLFA | 0.77 | 0.72 | 0.17 |
| Soil microbial community | Total bacterial PLFA | 0.76 | 0.71 | 0.18 |
| Soil microbial community | Total fungal PLFA | 0.98 | 0.95 | 0.19 |
| Root biomass allocation | Root dry weight | 0.81 | 0.81 | 0.05 |
| Root biomass allocation | Root surface area | 0.96 | 0.96 | 0.01 |
| Root biomass allocation | Root length | 0.92 | 0.92 | 0.03 |
| Soil properties | pH | 0.71 | 0.70 | 0.11 |
| Soil properties | NO_3_-N | 0.95 | 0.95 | 0.03 |
| Shoot biomass | Shoot dry weight | 1.00 | 1.00 | 0.00 |
| Ecosystem function | Net ecosystem exchange | 1.00 | 1.00 | 0.00 |

Methods S1, Table C: Original and bootstrapped path coefficients between all latent variables from the final model on net ecosystem exchange in the monoculture experiment (shown in Figure 3a).

| Latent variable/indicator |  | Latent variable/indicator | Original | Mean | SE |
| --- | --- | --- | --- | --- | --- |
| Leaf traits | -> | Root nutrients | -0.40 | -0.42 | 0.10 |
| Leaf traits | -> | Soil properties | 0.53 | 0.53 | 0.07 |
| Leaf traits | -> | Shoot dry weight | -0.41 | -0.41 | 0.10 |
| Root morphology | -> | Net ecosystem exchange | 0.19 | 0.20 | 0.07 |
| Root nutrients | -> | Soil microbial community | 0.27 | 0.26 | 0.11 |
| Root nutrients | -> | Root biomass | 0.30 | 0.31 | 0.08 |
| Root nutrients | -> | Shoot dry weight | -0.32 | -0.32 | 0.12 |
| Root nutrients | -> | Net ecosystem exchange | -0.18 | -0.19 | 0.07 |
| Soil microbial community | -> | Soil properties | -0.22 | -0.22 | 0.09 |
| Soil microbial community | -> | Shoot dry weight | 0.18 | 0.19 | 0.09 |
| Root biomass allocation | -> | Shoot dry weight | 0.37 | 0.37 | 0.09 |
| Soil properties | -> | Net ecosystem exchange | 0.22 | 0.23 | 0.08 |
| Shoot dry weight | -> | Net ecosystem exchange | -0.60 | -0.58 | 0.09 |

Methods S1, Table D: Original and bootstrapped loadings for each measured indicator for all latent variables from the final model on ecosystem respiration in the monoculture experiment (shown in Figure 3b).

| Latent variable | Measured indicator | Original | Mean | SE |
| --- | --- | --- | --- | --- |
| Leaf traits | Shoot N | 0.95 | 0.95 | 0.01 |
| Leaf traits | SLA | 0.78 | 0.77 | 0.06 |
| Leaf traits | C:N ratio | 0.90 | 0.91 | 0.01 |
| Root nutrients | Root N | 0.96 | 0.96 | 0.01 |
| Root nutrients | C:N ratio | 0.95 | 0.95 | 0.01 |
| Soil microbial community | Fungal to bacterial ratio (PLFA) | 0.79 | 0.80 | 0.07 |
| Soil microbial community | Gram negative PLFA | 0.76 | 0.72 | 0.15 |
| Soil microbial community | Total bacterial PLFA | 0.75 | 0.71 | 0.16 |
| Soil microbial community | Total fungal PLFA | 0.98 | 0.98 | 0.02 |
| Root biomass allocation | Root dry weight | 0.82 | 0.81 | 0.06 |
| Root biomass allocation | Root surface area | 0.96 | 0.96 | 0.01 |
| Root biomass allocation | Root length | 0.88 | 0.87 | 0.07 |
| Soil properties | pH | 0.67 | 0.66 | 0.11 |
| Soil properties | NO_3_-N | 0.97 | 0.97 | 0.01 |
| Shoot biomass | Shoot dry weight | 1.00 | 1.00 | 0.00 |
| Ecosystem function | Ecosystem respiration | 1.00 | 1.00 | 0.00 |

Methods S1, Table E: Original and bootstrapped path coefficients between all latent variables from the final model on ecosystem respiration in the monoculture experiment (shown in Figure 3b).

| Latent variable/indicator |  | Latent variable/indicator | Original | Mean | SE |
| --- | --- | --- | --- | --- | --- |
| Leaf traits | -> | Root nutrients | -0.42 | -0.41 | 0.11 |
| Leaf traits | -> | Soil properties | 0.55 | 0.56 | 0.06 |
| Leaf traits | -> | Shoot biomass | -0.42 | -0.42 | 0.08 |
| Root nutrients | -> | Soil microbial community | 0.28 | 0.29 | 0.09 |
| Root nutrients | -> | Root biomass allocation | 0.31 | 0.32 | 0.09 |
| Root nutrients | -> | Shoot dry weight | -0.31 | -0.33 | 0.09 |
| Soil microbial community | -> | Soil properties | -0.22 | -0.22 | 0.07 |
| Soil microbial community | -> | Shoot dry weight | 0.19 | 0.18 | 0.09 |
| Root biomass allocation | -> | Shoot dry weight | 0.36 | 0.37 | 0.08 |
| Shoot dry weight | -> | Ecosystem respiration | 0.45 | 0.44 | 0.08 |

Methods S1, Table F: Original and bootstrapped loadings for each measured indicators for all latent variables from the final model on net ecosystem exchange/ecosystem respiration in the community experiment (shown in Figure 4). Loadings are the same for both models, except the italic that are only present in the ecosystem respiration model.

| Latent variable | Measured indicator | Original | Mean | SE |
| --- | --- | --- | --- | --- |
| Leaf traits | Shoot N | 0.98 | 0.98. | 0.01 |
| Leaf traits | C:N ratio | 0.98 | 0.99 | 0.01 |
| Root morphology | SRL | 0.89 | 0.90 | 0.04 |
| Root morphology | Root diameter | 0.79 | 0.76 | 0.15 |
| Root nutrients | Root C | 0.63 | 0.55 | 0.25 |
| Root nutrients | C:N ratio | 0.77 | 0.76 | 0.18 |
| Root nutrients | Root N | 0.99 | 0.96 | 0.11 |
| Soil microbial community | C:N ratio | 0.68 | 0.66 | 0.09 |
| Soil microbial community | Total fungal PLFA | 0.96 | 0.97 | 0.01 |
| Soil microbial community | Total bacteria PLFA | 0.99 | 0.99 | 0.01 |
| Soil microbial community | Gram positive PLFA | 0.98 | 0.98 | 0.01 |
| Soil microbial community | Gram negative PLFA | 0.99 | 0.99 | 0.01 |
| Root biomass allocation | Root biomass | 0.95 | 0.96 | 0.01 |
| Root biomass allocation | Root:shoot ratio | 0.93 | 0.93 | 0.03 |
| Soil properties | Soil N | 0.96 | 0.96 | 0.04 |
| Soil properties | Soil C | 0.95 | 0.95 | 0.04 |
| Soil properties | NO_3_-N | 0.73 | 0.72 | 0.13 |
| *Shoot biomass* | *Shoot dry weight* | *1.00* | *1.00* | *0.00* |
| *Ecosystem function* | *Ecosystem respiration* | *1.00* | *1.00* | *0.00* |

Methods S1, Table G: Original and bootstrapped path coefficients between all latent variables from the final models on net ecosystem exchange and ecosystem respiration in the community experiment (shown in Figure 4). Path coefficients are the same for both models, except the italicized variables that are only present in the ecosystem respiration model.

| Latent variable/indicator |  | Latent variable/indicator | Original | Mean | SE |
| --- | --- | --- | --- | --- | --- |
| Leaf traits | -> | Root nutrients | 0.38 | 0.40 | 0.18 |
| Root morphology | -> | Root biomass allocation | 0.73 | 0.75 | 0.08 |
| Root nutrients | -> | Soil microbial community | 0.46 | 0.48 | 0.13 |
| Soil microbial community | -> | Soil properties | 0.41 | 0.45 | 0.11 |
| *Shoot dry weight* | -> | *Ecosystem respiration* | 0.36 | 0.36 | 0.12 |

Table S7. Regression matrix of plant leaf and root traits measured for 25 grassland species grown in monocultures. Values are *R*^2^ (*P*-values). Significant *p*-values (*P* ≤ 0.05) are shown in bold.

|  | LDMC^c^ | LFL^a^ | Shoot C^c^ | Shoot N^c^ | SLA^c^ | Shoot C to N ratio |
| --- | --- | --- | --- | --- | --- | --- |
| RDMC^f^ | 0.10 (**0.004)** | 0.02 (0.285) | 0.26 (**<0.001**) | 0.09 (**0.007**) | 0.06 (**0.025**) | 0.07 (**0.018**) |
| RFL^b^ | 0.12 (**0.005**) | 0.18 (**<0.001**) | 0.00 (0.927) | 0.01 (0.335) | 0.01 (0.309) | 0.04 (0.094) |
| Root C^d^ | 0.00 (0.775) | 0.02 (0.235) | 0.05 (0.053) | 0.07 (**0.017**) | 0.03 (0.122) | 0.07 (**0.019**) |
| Root diameter^f^ | 0.02 (0.267) | 0.37 (**<0.001**) | 0.00 (0.932) | 0.01 (0.422) | 0.01 (0.531) | 0.01 (0.392) |
| Root N ^d^ | 0.06 (**0.026**) | 0.08 (**0.017**) | 0.03 (0.127) | 0.22 (**<0.001**) | 0.02 (0.170) | 0.23 **(<0.001**) |
| SRL^e^ | 0.03 (0.138) | 0.27 (**<0.001**) | 0.04 (0.069) | 0.02 (0.175) | 0.01 (0.369) | 0.016 (0.271) |
| Root C to N ratio | 0.06 (**0.021**) | 0.09 (**0.012**) | 0.03 (0.145) | 0.37 **(<0.001**) | 0.02 (0.197) | 0.22 **(<0.001**) |

C = carbon, LFL = leaf fibre lignin, LDMC = leaf dry matter content, N = nitrogen, RFL = root fibre lignin, RDMC = root dry matter content, SLA = specific leaf area, SRL = specific root length.

All data ln(x) transformed before analysis. ^a^ n = 74; ^b^ n = 76; ^c^ n = 80; ^d^ n = 81; ^e^ n = 82 ^f^ n = 8

Table S8. Regression matrix of plant traits versus selected soil properties taken from 25 grassland species (n = 83) from contrasting functional groups (i.e., grasses, forbs, legumes) grown in monocultures. Soil properties that are shown had at least one significant relationship with at least one leaf or root trait. Soil properties with not significant relationships are not shown. Values are *R*^2^ (*P*-values). Significnat *P*-values (*P* ≤ 0.05) are shown in bold.

|  | LDMC | Shoot C | Shoot N | Shoot C:N | SLA | RDMC | Root C | Root N | Root C:N | Root diameter | SRL |
| --- | --- | --- | --- | --- | --- | --- | --- | --- | --- | --- | --- |
| Al | 0.03 (0.125) | 0.00 (0.974) | 0.05 (**0.037**) | 0.05 (0.051) | 0.01 (0.339) | 0.00 (0.783) | 0.00 (0.994) | 0.02 (0.215) | 0.02 (0.187) | 0.00 (0.537) | 0.01 (0.422) |
| DON | 0.00 (0.927) | 0.07 (**0.016**) | 0.06 (**0.032**) | 0.05 **(0.049)** | 0.03 (0.116) | 0.02 (0.203) | 0.01 (0.336) | 0.05 (**0.042**) | 0.08 **(0.011)** | 0.02 (0.174) | 0.00 (0.876) |
| Fungal to bacterial ratio (PLFA) | 0.01 (0.454) | 0.05 (0.059) | 0.09 (**0.010**) | 0.08 **(0.012)** | 0.10 (**0.005**) | 0.00 (0.964) | 0.00 (0.841) | 0.03 (0.110) | 0.03 (0.102) | 0.03 (0.156) | 0.02 (0.264) |
| Gram neg bacteria (PLFA) | 0.01 (0.306) | 0.09 (**0.010**) | 0.00 (0.694) | 0.01 (0.439) | 0.00 (0.639) | 0.00 (0.602) | 0.01 (0.348) | 0.00 (0.713) | 0.01 (0.488) | 0.00 (0.552) | 0.00 (0.659) |
| Gram pos bacteria (PLFA) | 0.01 (0.497) | 0.09 (**0.010**) | 0.00 (0.542) | 0.00 (0.571) | 0.01 (0.326) | 0.00 (0.905) | 0.00 (0.819) | 0.00 (0.623) | 0.01 (0.530) | 0.01 (0.515) | 0.00 (0.790) |
| K | 0.00 (0.651) | 0.00 (0.660) | 0.02 (0.199) | 0.02 (0.218) | 0.01 (0.350) | 0.01 (0.405) | 0.00 (0.737) | 0.01 (0.466) | 0.01 (0.348) | 0.03 (0.120) | 0.02 (0.216) |
| Mg | 0.01 (0.319) | 0.10 (**0.004**) | 0.12 (**0.002**) | 0.10 **(0.004)** | 0.09 (**0.009**) | 0.01 (0.405) | 0.01 (0.433) | 0.03 (0.144) | 0.03 (0.114) | 0.03 (0.120) | 0.00 (0.811) |
| Microbial biomass C | 0.05 (**0.048**) | 0.00 (0.580) | 0.01 (0.306) | 0.00 (0.541) | 0.00 (0.738) | 0.00 (0.601) | 0.00 (0.931) | 0.01 (0.450) | 0.01 (0.408) | 0.01 (0.311) | 0.03 (0.155) |
| Microbial biomass N | 0.05 (**0.049**) | 0.01 (0.415) | 0.00 (0.571 | 0.01 (0.432) | 0.05 (0.051) | 0.00 (0.773) | 0.00 (0.756) | 0.00 (0.603) | 0.00 (0.609) | 0.00 (0.895) | 0.02 (0.188) |
| NO_3_-N | 0.0 (0.600) | 0.17 (**<0.001**) | 0.48 (**<0.001**) | 0.47 **(<0.001)** | 0.19 (**<0.001**) | 0.10 (**0.004**) | 0.02 (0.212) | 0.21 (**<0.001**) | 0.17 **(<0.001)** | 0.01 (0.505) | 0.02 (0.270) |
| NO_3_-N (mineralisation rate) | 0.05 (**0.046**) | 0.00 (0.982) | 0.01 (0.485) | 0.01 (0.450) | 0.01 (0.403) | 0.00 (0.888) | 0.08 (**0.013**) | 0.2 (0.259) | 0.01 (0.406) | 0.06 (**0.031**) | 0.02 (0.227) |
| pH | 0.04 (0.092) | 0.00 (0.943) | 0.06 (**0.030**) | 0.07 **(0.020)** | 0.01 (0.308) | 0.00 (0.831) | 0.00 (0.888) | 0.02 (0.259) | 0.02 (0.181) | 0.00 (0.526) | 0.00 (0.418) |
| P (inorganic) | 0.02 (0.275) | 0.03 (0.146) | 0.05 (**0.047**) | 0.04 (0.082) | 0.06 (**0.033**) | 0.01 (0.315) | 0.01 (0.525) | 0.01 (0.514) | 0.01 (0.489) | 0.00 (0.742) | 0.0 (0.921) |
| TC (water extract) | 0.00 (0.912) | 0.00 (0.756) | 0.02 (0.177) | 0.05 (0.057) | 0.03 (0.136) | 0.00 (0.823) | 0.02 (0.169) | 0.01 (0.282) | 0.03 (0.098) | 0.00 (0.769) | 0.00 (0.806) |
| TIN | 0.00 (0.800) | 0.18 (**<0.001**) | 0.48 (**<0.001**) | 0.48 **(<0.001)** | 0.19 (**<0.001**) | 0.08 (**0.008**) | 0.03 (0.148) | 0.23 (**<0.001**) | 0.22 **(<0.001)** | 0.01 (0.369) | 0.01 (0.348) |
| TIN (mineralisation rate) | 0.06 (**0.034**) | 0.00 (0.691) | 0.01 (0.308) | 0.01 (0.344) | 0.01 (0.447) | 0.00 (0.973) | 0.06 (**0.026**) | 0.00 (0.695) | 0.00 (0.787) | 0.04 (0.089) | 0.00 (0.843) |
| TN | 0.00 (0.827) | 0.17 (**<0.001**) | 0.32 (**<0.001**) | 0.31 **(<0.001)** | 0.14 (**0.001**) | 0.07 (**0.015**) | 0.03 (0.152) | 0.18 (**<0.001**) | 0.18 **(<0.001)** | 0.03 (0.096) | 0.01 (0.332) |
| TOC (water extract) | 0.00 (0.686) | 0.01 (0.524) | 0.05 (**0.039**) | 0.05 (0.055) | 0.03 (0.158) | 0.00 (0.712) | 0.00 (0.754) | 0.02 (0.177) | 0.03 (0.129) | 0.00 (0.704) | 0.01 (0.364) |
| Total PLFA | 0.00 (0.740) | 0.05 (0.059) | 0.01 (0.536) | 0.00 (0.713) | 0.00 (0.655) | 0.00 (0.682) | 0.01 (0.368) | 0.00 (0.906) | 0.00 (0.613) | 0.01 (0.474) | 0.01 (0.319) |
| Total bacterial PLFA | 0.01 (0.330) | 0.10 (**0.006**) | 0.00 (0.627) | 0.00 (0.799) | 0.01 (0.509) | 0.00 (0.676) | 0.01 (0.450) | 0.00 (0.674) | 0.01 (0.502) | 0.01 (0.518) | 0.00 (0.677) |
| Total fungal PLFA | 0.00 (0.952) | 0.01 (0.494) | 0.07 (**0.024**) | 0.04 (0.069) | 0.08 (**0.013**) | 0.00 (0.767) | 0.00 (0.736) | 0.04 (0.065) | 0.03 (0.113) | 0.02 (0.261) | 0.01 (0.320) |

Al = aluminium, C = carbon, DON = dissolved organic nitrogen, K = potassium, Mg = magnesium, N = nitrogen, P = phosphorus, PLFA = phospholipid fatty acid, TC = total carbon, TIN = total inorganic nitrogen, TN = total nitrogen, TOC = total organic carbon

Fig. S6. Percentage cover and species richness of the target functional groups (grasses, forbs, legumes) added to 40 plots in a fully factorial design in temperate grassland in Selside, England. Within each panel, groups of eight bars topped with the same capital letters do not differ significantly at *P* ≤ 0.05 (Tukey’s HSD). When no letters are used, there is no significant difference among treatments. C = control, F = forb, L = legume, G = grass. Data shown as mean + SE and n = 5 for each treatment.

Table S9. Regression matrix of plant aboveground community weighted mean (CWM) traits versus belowground community traits measured on the 40 experimental community plots. Values are *R*^2^ (*P*-values) and significant values (*P* ≤ 0.05) are shown in bold.

|  | LDMC | Shoot C | Shoot C:N | Shoot N | SLA |
| --- | --- | --- | --- | --- | --- |
| Root C | 0.05 (0.219) | 0.10 (0.082) | 0.02 (0.478) | 0.00 (0.973) | 0.01 (0.590) |
| Root C:N | 0.00 (0.747) | 0.04 (0.260) | 0.04 (0.281) | 0.10 (0.073) | 0.01 (0.499) |
| Root N | 0.00 (0.855) | 0.01 (0.530) | 0.22 (0.055) | **0.12 (0.046)** | 0.02 (0.409) |
| Root diameter | 0.02 (0.491) | 0.00 (0.994) | 0.00 (0.919) | 0.00 (0.947) | 0.01 (0.686) |
| SRL | 0.00 (0.844) | 0.00 (0.791) | 0.02 (0.458) | 0.01 (0.567) | 0.03 (0.328) |

C = carbon, LFL = leaf fibre lignin, LDMC = leaf dry matter content, N = nitrogen, RFL = root fibre lignin, RDMC = root dry matter content, SLA = specific leaf area, SRL = specific root length.

Table S10. Regression matrix of plant traits versus soil abiotic and biotic properties measured on the 40 community plots. Values are R^2^ (*P*-values) and significant values (*P* ≤ 0.05) are shown in bold.

|  | LDMC | SLA | Shoot C | Shoot C:N | Shoot N | Root C | Root C:N | Root diameter | Root N | SRL |
| --- | --- | --- | --- | --- | --- | --- | --- | --- | --- | --- |
| DON | 0.00 (0.810) | 0.01 (0.534) | 0.03 (0.325) | 0.00 (0.753) | 0.02 (0.419) | 0.07 (0.131) | 0.02 (0.479) | 0.08 (0.102) | 0.00 (0.780) | 0.07 (0.125) |
| Fungal to bacterial ratio (PLFA) | 0.00 (0.777) | 0.01 (0.561) | 0.00 (0.704) | 0.06 (0.161) | 0.06 (0.188) | 0.08 (0.115) | 0.00 (0.731) | 0.02 (0.448) | 0.01 (0.541) | 0.04 (0.236) |
| Gram neg bacteria (PLFA) | 0.00 (0.935) | 0.00 (0.779) | 0.00 (0.725) | 0.08 (0.100) | 0.08 (0.121) | **0.16** **(0.021)** | 0.07 (0.136) | 0.04 (0.268) | **0.16** (**0.021)** | 0.00 (0.990) |
| Gram pos bacteria (PLFA) | 0.00 (0.999) | 0.01 (0.673) | 0.00 (0.799) | 0.09 (0.083) | 0.08 (0.110) | **0.18** **(0.015)** | 0.05 (0.234) | 0.04 (0.273) | **0.13** **(0.045)** | 0.00 (0.899) |
| Gram pos to gram neg ratio (PLFA) | 0.02 (0.486) | 0.07 (0.144) | 0.02 (0.464) | 0.01 (0.549) | 0.00 (0.886) | 0.02 (0.480) | **0.15** **(0.024)** | 0.00 (0.913) | 0.16 (0.026) | 0.02 (0.392) |
| IN (mineralisation rate) | 0.02 (0.414) | 0.02 (0.492) | 0.12 (0.052) | 0.00 (0.949) | 0.00 (0.841) | 0.02 (0.425) | 0.01 (0.645) | 0.01 (0.540) | 0.02 (0.484) | 0.01 (0.531) |
| Microbial biomass C | 0.00 (0.827) | 0.03 (0.335) | 0.01 (0.548) | 0.01 (0.629) | 0.00 (0.852) | 0.05 (0.230) | 0.06 (0.167) | 0.03 (0.340) | 0.09 (0.091) | 0.00 (0.720) |
| Microbial biomass N | 0.00 (0.964) | 0.01 (0.614) | 0.03 (0.338) | 0.01 (0.518) | 0.00 (0.707) | 0.06 (0.172) | 0.08 (0.108) | 0.05 (0.193) | **0.12** **(0.050)** | 0.02 (0.441) |
| Microbial biomass C:N | 0.04 (0.286) | 0.03 (0.325) | 0.05 (0.210) | 0.08 (0.122) | 0.05 (0.193) | 0.05 (0.198) | 0.06 (0.179) | 0.03 (0.375) | 0.08 (0.111) | 0.03 (0.315) |
| NH_4_-N | 0.02 (0.462) | 0.03 (0.354) | 0.06 (0.186) | **0.14 (0.035)** | 0.10 (0.070) | 0.02 (0.421) | 0.02 (0.416) | 0.00 (0.800) | 0.04 (0.257) | 0.00 (0.922) |
| NO_3_-N | 0.01 (0.554) | 0.00 (0.860) | 0.01 (0.562) | 0.05 (0.200) | 0.04 (0.276) | 0.03 (0.381) | 0.01 (0.590) | 0.00 (0.951) | 0.03 (0.340) | 0.02 (0.422) |
| ON (mineralisation rate) | 0.01 (0.669) | 0.01 (0.597) | 0.06 (0.178) | 0.00 (0.940) | 0.01 (0.506) | 0.06 (0.162) | 0.01 (0.669) | 0.06 (0.187) | 0.00 (0.950) | 0.06 (0.177) |
| pH | 0.03 (0.321) | 0.09 (0.085) | 0.02 (0.467) | 0.03 (0.363) | 0.05 (0.235) | 0.09 (0.087) | 0.00 (0.727) | 0.08 (0.113) | 0.00 (0.951) | 0.01 (0.520) |
| Soil C | 0.00 (0.882) | 0.04 (0.249) | 0.01 (0.606) | 0.00 (0.718) | 0.00 (0.956) | 0.07 (0.152) | 0.01 (0.580) | 0.01 (0.570) | 0.00 (0.953) | 0.01 (0.616) |
| Soil C:N | 0.00 (0.940) | 0.01 (0.645) | 0.04 (0.260) | 0.10 (0.076) | 0.06 (0.168) | 0.11 (0.059) | 0.00 (0.738) | 0.01 (0.533) | 0.02 (0.391) | 0.00 (0.829) |
| Soil N | 0.00 (0.873) | 0.03 (0.338) | 0.01 (0.507) | 0.02 (0.425) | 0.01 (0.655) | 0.09 (0.089) | 0.01 (0.691) | 0.02 (0.469) | 0.00 (0.881) | 0.01 (0.687) |
| TIN | 0.02 (0.417) | 0.03 (0.374) | 0.06 (0.178) | **0.15 (0.025)** | 0.11 (0.055) | 0.03 (0.346) | 0.02 (0.384) | 0.00 (0.828) | 0.05 (0.207) | 0.00 (0.922) |
| TN | 0.00 (0.700) | 0.00 (0.696) | 0.04 (0.238) | 0.00 (0.881) | 0.00 (0.720) | 0.08 (0.114) | 0.01 (0.636) | 0.07 (0.147) | 0.00 (0.998) | 0.06 (0.153) |
| TN (mineralisation rate) | 0.02 (0.442) | 0.00 (0.897) | **0.13 (0.042)** | 0.00 (0.933) | 0.01 (0.608) | 0.06 (0.186) | 0.00 (0.964) | 0.00 (0.714) | 0.01 (0.686) | 0.00 (0.708) |
| Total PLFA | 0.00 (0.885) | 0.00 (0.920) | 0.00 (0.730) | 0.11 (0.063) | 0.09 (0.086) | **0.18** **(0.017)** | 0.07 (0.139) | 0.04 (0.277) | **0.17** **(0.020)** | 0.00 (0.954) |
| Total bacterial PLFA | 0.00 (0.957) | 0.00 (0.746) | 0.00 (0.746) | 0.09 (0.094) | 0.08 (0.117) | **0.17** **(0.019)** | 0.06 (0.158) | 0.04 (0.268) | **0.15** **(0.026)** | 0.00 (0.962) |
| Total fungal PLFA | 0.00 (0.879) | 0.00 (0.701) | 0.00 (0.837) | 0.08 (0.116) | 0.07 (0.133) | **0.14** **(0.032)** | 0.08 (0.123) | 0.04 (0.288) | **0.17** (**0.019)** | 0.00 (0.856) |

C = carbon, DON = dissolved organic nitrogen, IN = inorganic nitrogen, LDMC = leaf dry matter content, N = nitrogen, ON = organic nitrogen, P = phosphorus, PLFA = phospholipid fatty acid, SLA = specific leaf area, SRL = specific root length, TC = total carbon, TIN = total inorganic nitrogen, TN = total nitrogen, TOC = total organic carbon

Fig. S7. Effect of plant species addition treatments on community weighted mean (CWM) shoot nitrogen (N) content. Data are means ± SE and n = 5 per treatment. Inset shows *F*-statistic and *p*-values from one-way ANOVA with block as a random factor. Bars topped with the same letter do not differ significantly at *P* ≤ 0.05 (Tukey’s HSD). C = control, F = forbs, G = grasses, L = legumes.

Table S11. Mean, standard deviation (SD), coefficient of variation (CV) and minimum and maximum values for functional traits, soil abiotic properties and the soil microbial community from the monoculture experiment. C = carbon, N = nitrogen, LDMC = leaf dry matter content, P = phosphorus, PLFA = phospholipid fatty acid, RDMC = root dry matter content, SLA = specific leaf area, SRL = specific root length.

| Variable | | Mean | ± | SD | CV | Min | Max |
| --- | --- | --- | --- | --- | --- | --- | --- |
| *Plant functional traits* | |  |  |  |  |  |  |
|  | Leaf C (%) | 43.61 | ± | 1.80 | 4.14 | 39.57 | 46.54 |
|  | Leaf N (%) | 2.62 | ± | 1.00 | 36.81 | 0.58 | 5.23 |
|  | Leaf C:N ratio | 19.62 | ± | 10.10 | 51.44 | 8.78 | 69.45 |
|  | SLA (mm^2^ mg^-1^) | 23.47 | ± | 6.90 | 29.20 | 10.74 | 45.61 |
|  | LDMC (mg g^-1^) | 209.98 | ± | 57.60 | 27.44 | 107.38 | 408.51 |
|  | Leaf lignin (%) | 3.21 | ± | 1.60 | 48.43 | 1.19 | 10.60 |
|  | Root C (%) | 45.43 | ± | 1.60 | 3.59 | 41.96 | 51.22 |
|  | Root N (%) | 0.97 | ± | 0.50 | 53.78 | 0.33 | 2.70 |
|  | Root C:N ratio | 57.00 | ± | 23.50 | 41.24 | 16.81 | 137.24 |
|  | SRL (m g^-1^) | 141.33 | ± | 105.70 | 74.78 | 11.42 | 449.14 |
|  | Average root diameter (mm) | 0.24 | ± | 0.10 | 32.73 | 0.14 | 0.60 |
|  | RDMC (mg g^-1^) | 162.92 | ± | 47.70 | 29.28 | 67.84 | 324.17 |
|  | Root lignin (%) | 8.84 | ± | 4.30 | 48.41 | 1.22 | 19.52 |
| *Plant productivity* | |  |  |  |  |  |  |
|  | Shoot biomass (g m^-2^) | 1129.87 | ± | 637.70 | 390.89 | 176.49 | 3031.46 |
|  | Root biomass (g m^-3^) | 954.96 | ± | 735.80 | 77.05 | 31.80 | 2917.32 |
| *Soil abiotic properties* | |  |  |  |  |  |  |
|  | pH | 4.71 | ± | 0.30 | 7.21 | 4.07 | 5.31 |
|  | Soil C (%) | 6.68 | ± | 0.70 | 10.66 | 5.39 | 8.52 |
|  | Soil N (%) | 0.68 | ± | 0.10 | 12.37 | 0.53 | 0.88 |
|  | Soil P (%) | 0.016 | ± | 0.001 | 19.06 | 0.011 | 0.023 |
|  | Total inorganic N (µg N g^-1^) | 8.46 | ± | 7.60 | 89.81 | 1.36 | 43.02 |
|  | Dissolved organic N (µg N g^-1^) | 14.60 | ± | 4.20 | 28.94 | 4.69 | 40.25 |
|  | Mineralisable inorganic N (µg N g^-1^) | 3.52 | ± | 1.10 | 31.97 | 1.45 | 6.01 |
| *Soil microbial community* | |  |  |  |  |  |  |
|  | Microbial biomass C (µC g^-1^) | 298.68 | ± | 141.90 | 47.50 | 45.00 | 608.00 |
|  | Microbial biomass N (µN g^-1^) | 65.42 | ± | 22.60 | 34.54 | 24.00 | 144.00 |
|  | Microbial biomass C:N | 0.92 | ± | 9.80 | 4.72 | 2.15 | 45.43 |
|  | Total PLFAs (µg g^-1^) | 195.09 | ± | 41.60 | 21.30 | 115.12 | 453.21 |
|  | Fungal PLFAs (µg g^-1^) | 4.04 | ± | 2.20 | 54.08 | 1.13 | 14.39 |
|  | Bacterial PLFAs (µg g^-1^) | 116.72 | ± | 21.30 | 18.27 | 85.18 | 256.68 |
|  | Fungal:bacterial ratio | 0.03 | ± | 0.02 | 43.13 | 0.01 | 0.08 |
|  | Gram positive bacterial PLFAs (µg g^-1^) | 37.72 | ± | 6.30 | 16.61 | 28.50 | 76.28 |
|  | Gram negative bacterial PLFAs (µg g^-1^) | 78.13 | ± | 15.60 | 19.96 | 51.50 | 178.43 |
|  | Gram positive:gram negative ratio | 0.49 | ± | 0.10 | 11.79 | 0.40 | 0.71 |

Table S12. Mean, standard deviation (SD), coefficient of variation (CV) and minimum and maximum values for plant functional traits, soil abiotic properties and the soil microbial community from the community experiment. C = carbon, CWM = community-weighted mean, N = nitrogen, LDMC = leaf dry matter content, P = phosphorus, PLFA = phospholipid fatty acid, RDMC = root dry matter content, SLA = specific leaf area, SRL = specific root length.

| Variable | | Mean | ± | SD | CV | Min | Max |
| --- | --- | --- | --- | --- | --- | --- | --- |
| *Plant functional traits* | |  |  |  |  |  |  |
|  | CWM leaf C (%) | 42.78 | ± | 0.35 | 0.82 | 41.71 | 43.55 |
|  | CWM leaf N (%) | 2.19 | ± | 0.14 | 6.25 | 1.97 | 2.54 |
|  | CWM leaf C:N ratio | 21.59 | ± | 1.13 | 5.23 | 18.96 | 23.52 |
|  | CWM SLA (mm^2^ mg^-1^) | 31.60 | ± | 28.23 | 8.93 | 25.79 | 41.73 |
|  | CWM LDMC (mg g^-1^) | 228.87 | ± | 16.32 | 7.13 | 205.93 | 295.96 |
|  | Root C (%) | 43.38 | ± | 2.23 | 5.13 | 32.11 | 45.53 |
|  | Root N (%) | 0.90 | ± | 0.08 | 9.20 | 0.63 | 1.10 |
|  | Root C:N ratio | 48.40 | ± | 3.69 | 7.62 | 39.64 | 58.35 |
|  | SRL (m g^-1^) | 72.35 | ± | 14.77 | 20.42 | 41.78 | 106.47 |
|  | Average root diameter (mm) | 0.37 | ± | 0.02 | 4.52 | 0.33 | 0.41 |
| *Plant productivity* | |  |  |  |  |  |  |
|  | Shoot biomass (g m^-2^) | 529.48 | ± | 122.50 | 23.13 | 352.12 | 925.36 |
|  | Root biomass (g m^-2^) | 349.74 | ± | 85.99 | 24.59 | 206.32 | 522.84 |
| *Plant functional diversity indices*^†^ | |  |  |  |  |  |  |
|  | Functional Richness (FRic) | 0.76 | ± | 0.49 | 64.82 | 0.03 | 1.84 |
|  | Functional Diversity (FDiv) | 0.75 | ± | 0.06 | 7.98 | 0.66 | 0.93 |
|  | Functional Evenness (FEve) | 0.71 | ± | 0.10 | 13.76 | 0.45 | 0.86 |
|  | Functional Dispersion (FDis) | 1.72 | ± | 0.16 | 9.11 | 1.30 | 2.07 |
|  | Rao’s Quadratic Entropy (RaoQ) | 3.30 | ± | 0.51 | 15.38 | 2.06 | 4.59 |
| *Soil abiotic properties* | |  |  |  |  |  |  |
|  | pH | 6.18 | ± | 0.19 | 3.09 | 5.94 | 6.79 |
|  | Soil C (%) | 4.96 | ± | 1.27 | 25.63 | 3.53 | 8.54 |
|  | Soil N (%) | 0.45 | ± | 0.13 | 27.81 | 0.32 | 0.83 |
|  | Soil P (%) | 0.030 | ± | 0.017 | 51.53 | 0.017 | 0.076 |
|  | Total inorganic N (µg N g^-1^) | 4.72 | ± | 1.48 | 31.43 | 2.75 | 9.04 |
|  | Dissolved organic N (µg N g^-1^) | 15.39 | ± | 5.39 | 35.04 | 7.53 | 26.10 |
|  | Mineralisable inorganic N (µg N g^-1^) | 1.06 | ± | 5.34 | 505.83 | -3.47 | 22.41 |
| *Soil microbial community* | |  |  |  |  |  |  |
|  | Microbial biomass C (µC g^-1^) | 488.35 | ± | 190.99 | 39.11 | 212.42 | 1168.14 |
|  | Microbial biomass N (µN g^-1^) | 444.25 | ± | 197.82 | 44.53 | 171.20 | 852.16 |
|  | Microbial biomass C:N | 1.18 | ± | 0.28 | 23.70 | 0.80 | 1.94 |
|  | Total PLFAs (µg g^-1^) | 47.80 | ± | 15.62 | 32.67 | 18.46 | 73.59 |
|  | Fungal PLFAs (µg g^-1^) | 11.56 | ± | 3.57 | 30.90 | 4.90 | 17.51 |
|  | Bacterial PLFAs (µg g^-1^) | 24.51 | ± | 7.99 | 32.62 | 10.06 | 37.95 |
|  | Fungal:bacterial ratio | 0.47 | ± | 0.03 | 6.20 | 0.41 | 0.56 |
|  | Gram positive bacterial PLFAs (µg g^-1^) | 6.59 | ± | 2.16 | 32.75 | 2.77 | 10.43 |
|  | Gram negative bacterial PLFAs (µg g^-1^) | 17.76 | ± | 5.79 | 32.62 | 7.22 | 27.25 |
|  | Gram positive:gram negative ratio | 0.37 | ± | 0.02 | 4.71 | 0.34 | 0.42 |

† Functional diversity indices were calculated using leaf traits with the dbFD function from the *FD* package in R (*v*1.0-12, Laliberté, Legendre and Shipley, 2014). These functional diversity indices are distance-based multidimensional metrics that integrate the values of multiple continuous leaf traits of the species occurring in each plot and, except FRic, are weighted by species relative abundance: FRic and FDiv are measured as convex hull volumes; FEve is measured as the regularity of occurrence and abundance of species along the minimum spanning tree; FDis and RaoQ measure average distance to the centroid (for details see cited literature in the documentation of the *FD* package).
